# The impact of the DREAMS partnership on HIV incidence among young women who sell sex in two Zimbabwean cities: results of a non-randomised study

**DOI:** 10.1136/bmjgh-2020-003892

**Published:** 2021-04-27

**Authors:** Sungai T Chabata, Bernadette Hensen, Tarisai Chiyaka, Phillis Mushati, Sithembile Musemburi, Jeffrey Dirawo, Joanna Busza, Sian Floyd, Isolde Birdthistle, James R Hargreaves, Frances M Cowan

**Affiliations:** 1 Centre for Sexual Health and HIV/AIDS Research (CeSHHAR) Zimbabwe, Harare, Zimbabwe; 2 Department of Public Health, Erasmus MC, University Medical Center Rotterdam, Rotterdam, The Netherlands; 3 Department of Clinical Research, London School of Hygiene & Tropical Medicine, London, UK; 4 Centre for Evaluation, London School of Hygiene & Tropical Medicine, London, UK; 5 Department of Infectious Disease Epidemiology, London School of Hygiene & Tropical Medicine, London, UK; 6 Department of Population Health, London School of Hygiene & Tropical Medicine, London, UK; 7 Department of International Public Health, Liverpool School of Tropical Medicine, Liverpool, UK

**Keywords:** epidemiology, prevention strategies, HIV, AIDS, intervention study

## Abstract

**Introduction:**

Young women who sell sex (YWSS) in Zimbabwe remain at high risk of HIV infection. Effective HIV prevention strategies are needed. Through support to access a combination of evidence-based interventions, including oral pre-exposure prophylaxis (PrEP), the Determined, Resilient, Empowered, AIDS-free, Mentored and Safe (DREAMS) partnership aimed to reduce new HIV infections among adolescent girls and young women by 40% over 24 months.

**Methods:**

Non-randomised ‘plausibility’ evaluation, powered to detect a 40% HIV incidence difference between DREAMS and non-DREAMS sites. Two large cities with DREAMS funding were included, and four smaller non-DREAMS towns for comparison. In all sites, YWSS were enrolled to a cohort through peer-referral. Women were followed up for 24 months. HIV seroconversion was the primary outcome, with secondary outcomes identified through a theory of change. Outcomes were compared between YWSS recruited in DREAMS cities and non-DREAMS towns, adjusting for individual-level confounders and HIV prevalence at enrolment.

**Results:**

From April to July 2017, 2431 women were enrolled, 1859 of whom were HIV negative at enrolment; 1019 of these women (54.8%) were followed up from March to May 2019 and included in endline analysis. Access to clinical services increased, but access to socioeconomic interventions promoted by DREAMS was limited. A total of 79 YWSS HIV seroconverted, with HIV incidence among YWSS in DREAMS cities lower (3.1/100 person-years) than in non-DREAMS towns (5.3/100 person-years). In prespecified adjusted analysis, HIV incidence was lower in DREAMS cities but with weak statistical evidence (adjusted rate ratio (RR)=0.68; 95% CI 0.40 to 1.19; p=0.18). Women in DREAMS cities were more likely to report ever and ongoing PrEP use, consistent condom use, fewer sexual partners and less intimate partner violence.

**Conclusion:**

It is plausible that DREAMS lowered HIV incidence among YWSS in two Zimbabwean cities, but our evaluation provides weak statistical evidence for impact and suggests any reduction in incidence was lower than the anticipated 40% decline. We identified changes to some important ‘pathways to impact’ variables, including condom use.

**Key questions**

**What is already known?**

- Young women who sell sex (YWSS) in Eastern and Southern Africa are at high risk of HIV.
- Available evidence shows that these women are less engaged with HIV prevention services.
- There are few evaluations of interventions for the broader population of YWSS.

**What are the new findings?**

- We found higher engagement with clinical services among women in the places where DREAMS investments were made and differences in pre-exposure prophylaxis (PrEP) use between women recruited in the Determined, Resilient, Empowered, AIDS-free, Mentored and Safe (DREAMS) cities and non-DREAMS towns, at 24-month follow-up.
- After 24 months of follow-up, HIV incidence was high in both groups, and lower in DREAMS cities but with weak statistical evidence of a difference between the two groups.
- We found evidence for an impact on some secondary outcomes, including condom use, violence and number of partners, and some lessons for implementation.
- Overall, YWSS reported little uptake of social protection services.
- Implementation challenges, including limited experience of implementing partners working with general populations in working with YWSS and barriers faced by YWSS in accessing these general population services, need to be addressed to improve coverage of combination HIV prevention services.

**What do the new findings imply?**

- The available evidence indicates the continued need to implement and evaluate combination HIV prevention services for YWSS, including women who do not identify as sex workers.
- Further efforts are needed to identify approaches that enhance access to social protection services among YWSS combined with delivery of biomedical interventions, including PrEP.

## Introduction

In Eastern and Southern Africa, adolescent girls and young women (AGYW) aged 15–24 accounted for 26% of all new HIV infections in 2018.^1^ Young women who sell sex (YWSS), including young female sex workers (FSW) and women who sell sex but do not identify as FSW, are at especially high risk because of their high numbers of sexual partners, constrained ability to negotiate condom use and high prevalence of other sexually transmitted infections.^2 3^ Poverty, poor access to healthcare, stigma and discrimination, and physical and sexual violence further compound this risk.^2 4^ Reducing HIV infections among YWSS requires interventions that address these multiple determinants of risk.

In 2015, the DREAMS (Determined, Resilient, Empowered, AIDS-free, Mentored and Safe) partnership was launched in ten sub-Saharan African countries, including Zimbabwe. Through financial and technical support for a combination of evidence-based interventions, DREAMS aimed to reduce new HIV infections among AGYW by 40% over 24 months.^5 6^ In Zimbabwe, interventions included HIV testing, contraception and condom provision as well as the offer of oral pre-exposure prophylaxis (PrEP), with linkage to the broader package of DREAMS services.

We evaluated whether offering HIV testing services and increasing the availability of PrEP to YWSS aged 18–24, combined with community mobilisation and social protection interventions supported by DREAMS, reduced the number of new HIV infections by 40% over 24 months, compared with HIV incidence among YWSS residing in areas where DREAMS investments were not available.

## Methods

### Study design and participants

We conducted a non-randomised ‘plausibility’ evaluation of the impact of DREAMS on HIV incidence and other secondary outcomes.^7^ Our ‘plausibility’ evaluation reflects that financial and practical issues limited the study size alongside other limitations such as the lack of randomisation.^5 7^ Our prespecified approach was to build the strength of the evidence base related to an impact of DREAMS on HIV incidence through combined analysis of outcome and qualitative process data.

The study was conducted with YWSS in two large cities and four smaller towns in Zimbabwe. The cities were selected purposively as locations where DREAMS funding was being provided. The four smaller towns were selected for comparison based on their similarity with the DREAMS cities according to data from the ‘Sisters with a Voice’ (Sisters) programme,^5^ a national HIV programme for sex workers. Sisters provides free, comprehensive HIV prevention and care services to FSW through a network of peer educators and clinics in line with WHO guidance, including: provision of free condoms and contraceptives, HIV testing, syndromic management of sexually transmitted infections (STIs), community empowerment and legal advice supported by outreach workers and peer educators. In the DREAMS cities and one non-DREAMS town, Sisters was delivered through static sites open 5 days a week; in three of the four non-DREAMS towns, Sisters was delivered through mobile clinics in which service provision was only available 1 day per week.

### The DREAMS interventions

**Figure 1:**
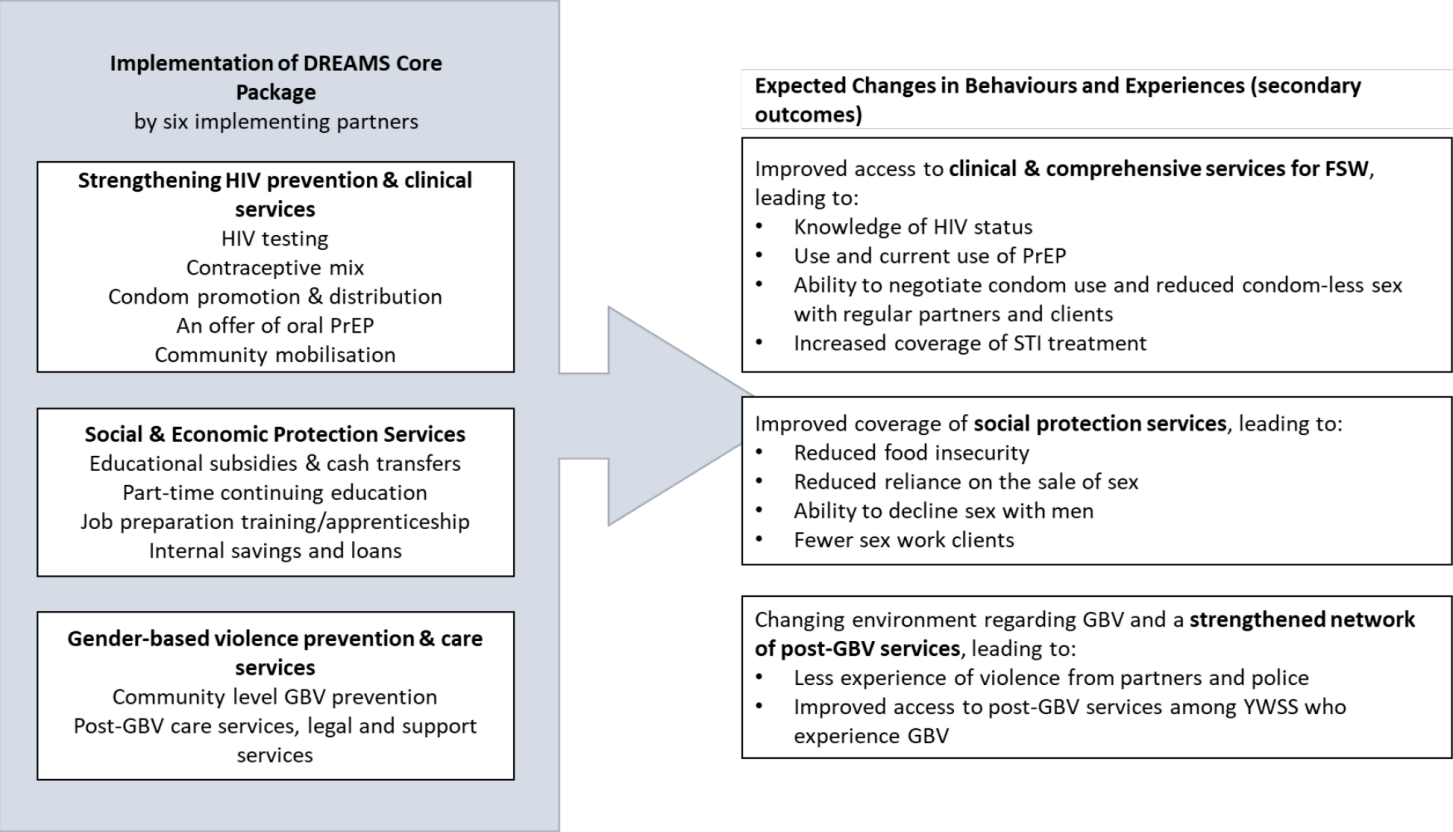
DREAMS core package of interventions and the expected changes in behaviours and experiences (secondary outcomes). DREAMS, Determined, Resilient, Empowered, AIDS-free, Mentored and Safe; FSW, female sex workers; GBV, gender-based violence; PrEP, pre-exposure prophylaxis; STI, sexually transmitted infection; YWSS, young women who sell sex.

In DREAMS cities, in addition to Sisters, a package of social and clinical interventions was available to YWSS. The DREAMS package was delivered through several implementing partners in the two cities; services available included social protection, life skills, education and vocational training, gender-based violence prevention and care, and HIV prevention, including condom promotion and distribution, an offer of PrEP combined with community empowerment and adherence support for those at highest risk of HIV (figure 1). Community-based activities aimed to increase demand for and uptake of PrEP and the DREAMS package more generally, and to support PrEP adherence. Entry into DREAMS could be through any implementing partner. In the non-DREAMS comparison towns YWSS could access usual care from the Sisters programme and the public health sector.

### Cohort recruitment and follow-up

Within each city/town we recruited individuals into the study cohort through peer-referral using the same approach to respondent-driven sampling (RDS) we have used in previous surveys.^2 5^ YWSS were eligible to participate if they were aged 18–24, had exchanged sex for money and/or material support in the past month, with it explicitly stated that sex acts with men would not have happened in the absence of an exchange, and if they were not planning to move from the site within the next 6 months.

We conducted community mapping to identify hotspots where young women sell sex to select ‘seed’ participants.^8^ Six seed participants were selected in each non-DREAMS town and 10 in each DREAMS city. Seed participants were given two coupons to recruit their peers. This process continued until the desired sample size was attained across both study groups. We previously described the participants enrolled at baseline, including details of the peer referral process.^2^ A summary of these and some additional analyses are reported in online supplemental appendix 5.

10.1136/bmjgh-2020-003892.supp1 Supplementary data

In both groups, YWSS were interviewed and offered HIV testing using a rapid HIV test at enrolment into the study in 2017. Budgetary constraints meant that women recruited in DREAMS cities were followed up at 12 months and 24 months after the initial enrolment survey, with women in non-DREAMS towns followed up at 24 months only. In 2019, at 24-month follow-up, study participants were re-interviewed and offered HIV testing as before. Complementary strategies were implemented to retain women in the study in the DREAMS and non-DREAMS groups, including WhatsApp messaging, outreach activities and engaging YWSS who recruited women to the study to inform women about the study follow-up.

### HIV testing procedures

At enrolment and follow-up, women were offered counselling and HIV testing services using a serial HIV testing algorithm adapted from Zimbabwe's national testing and counselling guidelines. Determine HIV-1/2 or First Response HIV-1-2 kit antibody testing was used as the first screening test. Where the result was HIV positive, this was confirmed using First Response HIV-1-2 kit or Determine HIV-1/2. Where the two test results were discordant, repeat testing was advised within 2 weeks. All women were given the results of their HIV test.

### Primary and secondary outcomes

The primary outcome was incident HIV infection over the 24-month study period. The outcome was calculated as the number of new HIV infections divided by the total person-years of follow-up during the 24-month study period, among YWSS who tested HIV-negative at enrolment. Selection of secondary outcomes reflected the hypothesised causal pathway for how DREAMS would reduce HIV incidence (figure 1).^5^ We assessed thirteen prespecified, self-reported, secondary outcomes including condom-less sex with regular partners and clients, knowledge of HIV status of themselves and their partners, whether selling sex was the primary means by which women support themselves, number of recent clients and food insecurity.

As part of a broader process evaluation, we collected qualitative data at three time points during the trial from implementing partner staff and participants with varying levels of engagement in different DREAMS interventions. We conducted semi-structured interviews with 14 implementing partner staff and 23 participants that explored perceptions and experiences of delivering or receiving DREAMS activities. We interviewed a further 17 participants 2–3 times to identify barriers and facilitators to engaging with the programme over time.

### Statistical analysis

In line with DREAMS targets, the study was powered to detect a 40% or greater reduction in HIV incidence after 24 months, assuming that HIV incidence in the comparison group would be 5.0%–8.0% per annum, as suggested by previous studies in Zimbabwe.^5^ Our analysis followed a prespecified analysis plan (online supplemental appendix 1).

We compared sociodemographic characteristics of enrolled YWSS by site and study group, including HIV status. We presented a flow diagram to illustrate enrolment and retention in the study at 24-month follow-up, and examined patterns of retention by age, marital status, highest level of education attained and whether women self-identified as FSW, all measured at enrolment. Using data at 24-month follow-up, we described self-reported uptake of DREAMS-related services in all sites and used logistic regression adjusted for age and site to compare uptake between groups.

Reflecting the non-randomised study design, we identified potential confounding factors to adjust for to obtain the fairest comparisons between the two study groups at endline. Critically, HIV prevalence at enrolment differed between the two study groups. Using factors considered a priori as associated with HIV prevalence at enrolment or likely to be associated with HIV incidence,^2 5^ including age, educational attainment, marital status, self-identification as a FSW, STI symptoms and number of sexual partners in the past month, we used logistic regression to model predictors of HIV prevalence at enrolment. We found that these factors, which differed between the two study groups, were associated with HIV prevalence. Adjusting for these factors reduced the difference in HIV prevalence at enrolment across both study groups. We therefore decided a priori to adjust for these individual-level factors in our primary analysis and to add a linear term for community-level HIV prevalence.

Primary analysis was based on the intention-to-treat principle, comparing cohorts recruited in the two DREAMS cities and four non-DREAMS towns regardless of participants’ individual-level engagement with services. We calculated HIV incidence and the prevalence of secondary outcome measures for each study site. We pooled the data across study sites and used Poisson regression with follow-up time estimated as the time between interview dates, or half of this for women who seroconverted. We first conducted an age-adjusted analysis comparing HIV incidence between the DREAMS cities and non-DREAMS towns, and subsequently conducted a fully adjusted analysis including the confounders listed above.

For secondary outcomes, we used logistic regression adjusted for confounders listed above and for each respective secondary outcome measured at enrolment. In these analyses, where <5 women reported the outcome in a site, we used Fisher’s exact test.

Our primary analysis excluded data collected from women followed up at 12 months post-enrolment in the DREAMS cities. In sensitivity analysis, we included this data. If a woman seroconverted by the 12-month follow-up survey, we placed the seroconversion date at the mid-point between enrolment and 12 months. If a woman seroconverted between the 12-month and 24-month follow-up surveys, we placed the seroconversion date at the mid-point between these surveys.

In a second sensitivity analysis, we applied the principles of the RDS-II method, dropping seed participants from the analysis and weighting responses for each woman by the inverse of her self-reported network size at baseline (ie, the number of other YWSS she could have recruited to the study).^9^ In our protocol paper, we described that a full statistical analysis plan would be developed before unblinding the data, but also suggested that our analysis would apply RDS weighting.^5^ During the development of the plan, but before looking at the data, we decided against the use of weighting in the primary analysis for the following reasons: the aim of our approach was to recruit balanced cohorts of YWSS in DREAMS and non-DREAMS sites and pool these for analysis, not to generate statistics representative of the cities or towns; the primary analysis was to be conducted only among the subset of women who were HIV-negative at enrolment and followed up successfully and therefore weighting may not be appropriate; and an unweighted analysis is simpler and more transparent, and is recommended in Poisson regression analyses of RDS data.^10^

Thirdly, assuming a uniform distribution and using 50 imputations, we randomly imputed the seroconversion date as a fraction of the interval between enrolment and 24-month survey dates, among women who seroconverted by the 24-month follow-up survey.^11 12^

### Patient and public involvement

FSW work closely with the research team at the Centre for Sexual Health and HIV/AIDS Research, Zimbabwe, however, were not directly involved in the development of the research questions and design of the study. FSW helped design and implement social mapping to identify YWSS hotspots to guide survey processes and to support RDS. YWSS were integrally involved in running all aspects of the DREAMS implementation for YWSS.

### Role of funding source

The Bill & Melinda Gates Foundation staff reviewed and provided non-binding comments on the protocol and study analysis plan, but had no role in data collection, data analysis, data interpretation or writing of the report. The corresponding author had full access to all the data in the study and had final responsibility for the decision to submit for publication.

## Results

### Study participants

**Figure 2:**
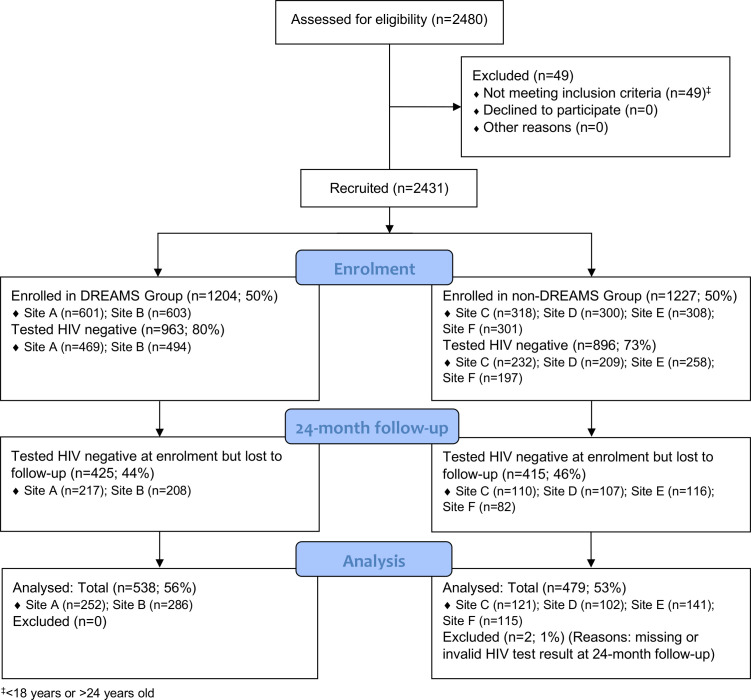
Consolidated Standards of Reporting Trials flow diagram of young women who sell sex recruited to the study and followed up at 24 months post-enrolment. DREAMS, Determined, Resilient, Empowered, AIDS-free, Mentored and Safe.

Between April and July 2017, we enrolled 2431 women (1204 in DREAMS cities and 1227 in non-DREAMS towns). Nineteen women had missing HIV test data. Among the remaining women, 76% (n=1859) tested HIV negative at enrolment (963, 80.0%, in DREAMS cities and 896, 73.0%, in non-DREAMS towns; figure 2). HIV prevalence was higher in the four non-DREAMS towns compared with the two DREAMS cities (26.3%, n=320 vs 19.5%, n=234; table 1 & online supplemental appendix 2 table 1).

**Table 1:** Key demographic and behavioural characteristics at enrolment of young women who sell sex testing HIV negative by group, 2017

	DREAMS cities (N=963)	Non-DREAMS towns (N=896)	Comparison p value
	n/N (%)	n/N (%)	
Age at recruitment			0.076
18–19	361/963 (37.5)	372/896 (41.5)	
20–24	602/963 (62.5)	524/896 (58.5)	
Highest level of education			<0.001
None/incomplete primary	28/963 (2.9)	77/896 (8.6)	
Complete primary	61/963 (6.3)	92/896 (10.3)	
Incomplete secondary	817/963 (84.8)	707/896 (78.9)	
Complete secondary or higher	57/963 (5.9)	20/896 (2.2)	
Marital status			<0.001
Single/never married	668/963 (69.4)	497/896 (55.5)	
Married/living together as if married	21/963 (2.2)	16/896 (1.8)	
Divorced/separated	270/963 (28.0)	379/896 (42.3)	
Widowed	4/963 (0.4)	4/896 (0.4)	
Years selling sex			0.001
0–2	508/962 (52.8)	538/893 (60.2)	
3+	454/962 (47.2)	355/893 (39.8)	
Self-identification as sex worker			0.847
No	319/952 (33.5)	302/890 (33.9)	
Yes	633/952 (66.5)	588/890 (66.1)	
Condom use at last with regular partner			0.002
No	252/739 (34.1)	294/696 (42.2)	
Yes	487/739 (65.9)	402/696 (57.8)	
Condom-less sex with regular partner in the past month			0.478
No	415/740 (56.1)	379/699 (54.2)	
Yes	325/740 (43.9)	320/699 (45.8)	
Condom use at last with client			0.477
No	86/743 (11.6)	65/627 (10.4)	
Yes	657/743 (88.4)	562/627 (89.6)	
Condom-less sex with client in the past month			0.001
No	611/745 (82.0)	555/628 (88.4)	
Yes	134/745 (18.0)	73/628 (11.6)	
STI symptoms in the last 12 months			0.067
No	775/963 (80.5)	690/896 (77.0)	
Yes	188/963 (19.5)	206/896 (23.0)	
Risk of common mental disorder			<0.001
No	595/963 (61.8)	624/896 (69.6)	
Yes	368/963 (38.2)	272/896 (30.4)	

DREAMS, Determined, Resilient, Empowered, AIDS-free, Mentored and Safe; STI, sexually transmitted infection.

Among women testing HIV negative at enrolment, women recruited in the DREAMS cities reported higher levels of educational attainment, were less likely to be separated/divorced and a higher proportion reported selling sex for ≥3 years compared with women in the non-DREAMS towns (table 1). Women in non-DREAMS towns reported lower levels of condom-less sex with clients and condom use with regular partners at last sex (online supplemental appendix 2 tables 1 and 2).

**Table 2:** Uptake of services available through the DREAMS partnership, by study group

	DREAMS cities (N=538)	Non-DREAMS towns (N=481)	DREAMS vs non-DREAMS	
	n/N (%)	n/N (%)	OR (95% CI)	P value
**Direct HIV prevention and clinical services**				
Recently HIV tested (within 6 months prior to the survey)				
No	181/537 (33.7)	152/478 (31.8)		
Yes	356/537 (66.3)	326/478 (68.2)	1.32 (0.83 to 2.10)	0.237*
Ever been offered PrEP				
No	285/538 (53.0)	476/481 (99.0)		
Yes	253/538 (47.0)	5/481 (0.9)	–	<0.001†
Current use of contraceptive methods (including condom)				
No	61/495 (12.3)	101/432 (23.4)		
Yes	434/495 (87.7)	331/432 (76.6)	1.37 (0.71 to 2.63)	0.343*
Attendance to Sisters with a Voice clinic in past 12 months				
No	221/538 (41.1)	344/480 (71.7)		
Yes	317/538 (58.9)	136/480 (28.3)	12.51 (6.90 to 22.69)	<0.001*
Saw condom promotion activities in the past 12 months				
No	175/536 (32.6)	225/479 (47.0)		
Yes	361/536 (67.4)	254/479 (53.0)	1.91 (1.22 to 3.00)	0.005*
Attendance to Sisters with a Voice community mobilisation meeting in past 12 months				
No	464/537 (86.4)	453/480 (94.4)		
Yes	73/537 (13.6)	27/480 (5.6)	22.76 (3.09 to 167.71)	0.002*
**Social and economic protection services**				
Receipt of cash transfer or educational subsidy in past 12 months				
No	516/538 (95.9)	480/480 (100.0)		
Yes	22/538 (4.1)	0/480 (0.0)	–	<0.001†
Participation in continuing education programme in past 12 months				
No	528/538 (98.1)	480/480 (100.0)		
Yes	10/538 (1.9)	0/480 (0.0)	–	0.002†
Participation in job preparation training in past 12 months				
No	529/538 (98.3)	480/480 (100.0)		
Yes	9/538 (1.7)	0/480 (0.0)	–	0.004†
Participation in apprenticeship in past 12 months‡				
No	538/538 (100.0)	480/480 (100.0)		
Yes	0/538 (0.0)	0/480 (0.0)	–	–
Participation in internal savings and loan group in past 12 months				
No	514/537 (95.7)	479/479 (100.0)		
Yes	23/537 (4.3)	0/479 (0.0)	–	<0.001†
**Gender-based violence care and support services**				
Accessed healthcare services after experiencing GBV in past 12 months§				
No	49/63 (77.8)	40/48 (83.3)		
Yes	14/63 (22.2)	8/48 (16.7)	1.38 (0.15 to 10.05)	0.832*
Provided with shelter in past 12 months (among women experiencing GBV)				
No	188/189 (99.5)	181/183 (98.9)		
Yes	1/189 (0.5)	2/183 (1.1)	–	0.549†

*Age and site adjusted Wald test p value.

†Fisher’s exact p value—OR and 95% CI could not be estimated using logistic regression due to sparse data.

‡Fisher’s exact p value or OR and 95% CI could not be estimated due to sparse data.

§Among YWSS who experienced sexual violence.

DREAMS, Determined, Resilient, Empowered, AIDS-free, Mentored and Safe; GBV, gender-based violence; PrEP, pre-exposure prophylaxis.

### Participant retention and follow-up

At 24-month follow-up, 1019 (54.8%) women testing HIV negative at enrolment were retained in the study (55.9%, n=538 in DREAMS cities; 53.7%, n=481 in non-DREAMS towns; figure 2). The mean follow-up time across all sites was 1.86 person-years (range in DREAMS cities: 1.76–1.90 person-years; non-DREAMS towns range: 1.83–1.97 person-years).

Enrolment characteristics and behaviours across the two groups were similar among women retained at 24-month follow-up (online supplemental appendix 2 table 3). Retained women were similar to women lost to follow-up in terms of marital status, educational attainment and whether they self-identified as FSW or not at enrolment (online supplemental appendix 3 table 4). In non-DREAMS towns, a lower proportion of YWSS aged 18–19 were retained (49.7%, n=185) compared with DREAMS cities (57.6%, n=208; p=0.046).

### Uptake of services available through the DREAMS partnership

Uptake of and access to clinical services were higher in DREAMS cities than non-DREAMS towns (table 2). In DREAMS cities, women were more likely to report accessing clinics targeting FSW (58.9%, n=317/538 vs 28.3%, n=136/480), seeing condom promotion activities (67.4%, n=361/536 vs 53.0%, n=254/479) and attending community mobilisation activities (13.6%, n=73/537 vs 5.6%, n=27/480).

Participation in savings and loans groups and cash transfers was low, with little difference between women in DREAMS cities and non-DREAMS towns (table 2). In qualitative data, we found that this partly reflected delays in establishing the YWSS component of the programme. In the early stages, service providers struggled to understand the specific needs of YWSS and felt uncomfortable integrating them with other youth. Many social programmes were fully enrolled prior to YWSS seeking to join, as implementing partners reached targets quickly from the broader population of young women. Over time, additional places were made available and prioritised for YWSS, although those delivering the services did not always have experience of working with this vulnerable group:

I think that was one of the issues that really made it difficult for us in terms of layering services because you see that there was much demand within our cohort of clients that would have wanted that service, but then that service was not available. … and issues to do with adolescents and young women selling sex are on the sensitive side. If you don’t have the skills, you will not be able to identify a large group of them [to recruit into the programme]. (Implementing Partner, Site A)

When YWSS did engage in opportunities, they sometimes felt stigmatised. We received reports of returning school pupils being publicly identified as ‘sex workers,’ denied access to the library, or made to sit outside classrooms and observe teaching through open windows:

Some [other children] will be saying, ‘why have you come to school? Why did you leave your work of selling sex?’ to make a scene in front of other people. … at times I felt so ashamed and I would feel it’s better not to go there. (18 years, Site B)

Other barriers stemmed from YWSS’ entrenched poverty, which meant they could not easily devote time to their studies, needing to earn money or look after family members. There were also additional costs such as bus fares and purchasing supplies for vocational training that YWSS could not afford. Other barriers to uptake included competing demands on YWSS’ time, that is, caring for siblings, and their inability to pay for costs associated with participation, such as transport to classes or uniforms and equipment required for vocational training. These challenges meant that YWSS had limited ability to access ‘layered’ services as intended.

### HIV incidence rate by study group and site

Overall, 79 YWSS HIV seroconverted after 24 months. HIV incidence was 3.60/100 person-years in DREAMS Site A and 2.76/100 person-years in DREAMS Site B. In non-DREAMS towns, the rate of new infections ranged from 4.28/100 person-years to 7.07/100 person-years (table 3).

**Table 3:** HIV incidence among young women who sell sex testing HIV negative at enrolment, by arm (A) and site (B) (N=1017)

	Number of seroconversions/person-years of follow-up	Rate per 100 person-years (95% CI)	Age-adjusted rate ratio (95% CI) p value	Fully adjusted rate ratio (95% CI)* p value
**(A) Comparison of HIV incidence among YWSS testing HIV negative at enrolment, by arm**				
Non-DREAMS (N=479)	48/907.62	5.29 (3.99 to 7.02)	1.0	1.0
DREAMS (N=538)	31/988.14	3.14 (2.21 to 4.46)	0.59 (0.38 to 0.93) p=0.022	0.68 (0.40 to 1.19) p=0.176
**(B) Comparison of HIV incidence among YWSS testing HIV negative at enrolment, by site**				
DREAMS Site A (n=252)	16/444.74	3.60 (2.20 to 5.87)	1.0	
DREAMS Site B (n=286)	15/543.40	2.76 (1.66 to 4.58)	0.75 (0.37 to 1.52) p=0.420	0.68 (0.33 to 1.43) p=0.313
Non-DREAMS Site C (n=121)	16/226.24	7.07 (4.33 to 11.54)	1.93 (0.96 to 3.88) p=0.063	1.56 (0.74 to 3.29) p=0.243
Non-DREAMS Site D (n=102)	11/192.90	5.70 (3.16 to 10.30)	1.57 (0.73 to 3.38) p=0.252	1.33 (0.58 to 3.03) p=0.503
Non-DREAMS Site E (n=141)	12/278.41	4.31 (2.45 to 7.59)	1.21 (0.57 to 2.56) p=0.617	1.04 (0.46 to 2.38) p=0.923
Non-DREAMS Site F (n=115)	9/210.07	4.28 (2.23 to 8.23)	1.16 (0.51 to 2.63) p=0.723	1.00 (0.43 to 2.34) p=0.996

*Adjusted for age, highest level of education attained, marital status, self-identification as female sex workers, STI symptoms, number of sexual partners in the past month, HIV prevalence (measured at enrolment).

DREAMS, Determined, Resilient, Empowered, AIDS-free, Mentored and Safe; STI, sexually transmitted infection; YWSS, young women who sell sex.

HIV incidence among YWSS in DREAMS cities was 3.1/100 person-years compared with 5.3/100 person-years among YWSS in non-DREAMS towns (RR=0.59; 95% CI 0.38 to 0.93). In our primary analysis, adjusting for confounding variables, HIV incidence was lower in DREAMS cities than in non-DREAMS towns, but with weak statistical evidence of a difference (adjusted rate ratio (RR)=0.68; 95% CI 0.40 to 1.19; p=0.18) (table 3).

In a sensitivity analysis, including data from women in DREAMS cities followed up at 12 months, we found a very similar result (adjusted RR=0.79, 95% CI 0.47 to 1.32; p=0.36; online supplemental appendix 6 table 8). When we applied the principles of RDS-II methodology, the intervention effect was larger, the point estimate exceeded the 40% incidence reduction hypothesised by DREAMS, and the result was of borderline statistical significance (adjusted RR=0.55, 95% CI 0.28 to 1.08; p=0.08; online supplemental appendix 6 table 9). Randomly imputing the seroconversion date gave results identical to using the mid-point as seroconversion date (online supplemental appendix 6 table 10).

### Secondary outcomes by study group and site

Compared with women in the non-DREAMS towns, YWSS in the DREAMS cities were more likely to report ever having used PrEP (28.1%, n=151/538 vs 0.6%, n=2/481; table 4). In DREAMS cities, 11.5% (62/538) of YWSS reported current use of PrEP at 24 months, with no YWSS in non-DREAMS towns reporting current PrEP use. Despite higher self-reported use of PrEP in DREAMS cities, we found that within those cities there were a similar percentage of HIV infections between YWSS who did and did not report having ever used PrEP (4.6%, n=7/151 vs 6.2%, n=24/387) and current use of PrEP was low. Additionally, PrEP use among YWSS who reported condom-less sex with a client or regular partner in the past month was very low (11.4%, n=31/273).

**Table 4:** Comparison of DREAMS secondary outcomes between the two DREAMS cities and the four non-DREAMS comparison towns, 2019

	DREAMS cities	Non-DREAMS towns	DREAMS vs non-DREAMS			
	(N=538)	(N=481)	Age-adjusted		Fully adjusted*	
	n/N (%)	n/N (%)	OR (95% CI)	P value	OR (95% CI)	P value
**Improved access to clinical services and HIV prevention services**						
Knowledge of HIV status						
No	119/538 (22.1)	118/481 (24.5)				
Yes	419/538 (77.9)	363/481 (75.5)	1.15 (0.86 to 1.54)	0.358	1.22 (0.90 to 1.66)	0.197
Ever taken PrEP						
No	378/538 (71.9)	478/481 (99.4)				
Yes	151/538 (28.1)	3/481 (0.6)	62.22 (19.69 to 196.62)	<0.001	**63.82 (19.78 to 205.90**)	**<0.001**
Ability to negotiate condom use with any partner						
No	40/538 (7.4)	91/480 (19.0)				
Yes	498/538 (92.6)	389/480 (81.0)	2.92 (1.97 to 4.34)	<0.001	**3.39 (2.24 to 5.14**)	**<0.001**
Knowledge of the HIV status of at least one of their three most recent partners						
No	179/528 (33.9)	210/474 (44.3)				
Yes	349/528 (66.1)	264/474 (55.7)	1.55 (1.20 to 2.00)	0.001	**1.38 (1.06 to 1.81**)	**0.018**
Condom-less sex with regular partner in the past month						
No	275/536 (51.3)	199/478 (41.6)				
Yes	261/536 (48.7)	294/478 (58.4)	0.68 (0.53 to 0.87)	0.002	**0.72 (0.53 to 0.98**)	**0.034**
Condom-less sex with client in the past month						
No	475/535 (88.8)	396/478 (82.8)				
Yes	60/535 (11.2)	82/478 (17.2)	0.61 (0.43 to 0.87)	0.007	**0.58 (0.38 to 0.89**)	**0.013**
Accessed STI treatment services in the past 12 months†						
No	7/74 (9.5)	18/93 (19.4)				
Yes	67/74 (90.5)	75/93 (80.6)	2.36 (0.92 to 6.03)	0.073	–	–
**Improved coverage of social and economic protection service**						
Food insecurity						
No	367/538 (68.2)	295/479 (61.6)				
Yes	171/538 (31.8)	184/479 (38.4)	0.75 (0.58 to 0.97)	0.027	0.81 (0.61 to 1.07)	0.130
Selling sex is the main way to support myself						
No	196/538 (36.4)	159/479 (33.2)				
Yes	342/538 (63.6)	320/479 (66.8)	0.87 (0.67 to 1.13)	0.284	0.93 (0.71 to 1.22)	0.585
Ever been unable to decline sex in the past month						
Never/not in the past month	257/534 (48.1)	236/474 (49.8)				
At least once in the past month	277/534 (51.9)	238/474 (50.2)	1.07 (0.83 to 1.37)	0.595	1.05 (0.81 to 1.36)	0.689
Number of sex work clients in the past month						
≤3	283/537 (52.7)	203/468 (43.4)				
>3	254/537 (47.3)	265/468 (56.6)	0.68 (0.53 to 0.88)	0.003	**0.66 (0.50 to 0.87**)	**0.003**
**Gender-based violence prevention, and care and support services**						
Experience of violence from partners in the past 12 months						
No	434/538 (80.7)	345/481 (71.7)				
Yes	104/538 (19.3)	136/481 (28.3)	0.61 (0.45 to 0.81)	0.001	**0.64 (0.47 to 0.87**)	**0.005**
Experience of violence from police in the past 12 months						
No	531/538 (98.7)	473/479 (98.7)				
Yes	7/538 (1.3)	6/479 (1.3)	1.04 (0.35 to 3.11)	0.070	1.01 (0.32 to 3.16)	0.982

Bold values highlight significant results.

*Adjusted for age, highest level of education attained, marital status, self-identification as female sex workers (measured at baseline), and for each respective secondary outcome measured at enrolment.

†Adjusted OR and 95% CI could not be estimated using logistic regression due to sparse data.

DREAMS, Determined, Resilient, Empowered, AIDS-free, Mentored and Safe; PrEP, pre-exposure prophylaxis; STI, sexually transmitted infection.

More women in the DREAMS cities reported the ability to negotiate condom use (92.6%; n=498/538 vs 81.0%, n=389/480; adjusted OR=3.39 95% CI 2.24 to 5.14; table 4). Women in DREAMS cities were less likely to report condom-less sex with clients (11.2%, n=60/535 vs 17.2%, n=82/478; adjusted OR=0.58 95% CI 0.38 to 0.89) and having had more than three clients in the past month (47.3%, n=254/537 vs 56.6%, n=265/468; adjusted OR=0.66 95% CI 0.50 to 0.87). Fewer women in DREAMS cities reported experiencing violence from partners in the past 12 months (19.3%, n=104/538 vs 28.3%, n=136/481; adjusted OR=0.64 95% CI 0.47 to 0.87). We found little evidence for differences in other secondary outcomes, including whether the YWSS’ primary means of support was sex work, and their ability to decline sex.

Secondary outcomes varied across sites and, for some outcomes, were not always in the direction expected (online supplemental appendix 4 table 5). For example, condom-less sex with client in the past month in two non-DREAMS towns (8.0%, n=8/100 and 10.7%, n=13/122) was lower or similar to levels reported by women in DREAMS cities. Similarly, the ability to negotiate condom use in these two non-DREAMS towns (92.6% n=113/122 and 85.3% n=87/102) was higher than or similar to levels reported in each DREAMS city.

## Discussion

After 24 months of follow-up, we found that HIV incidence was lower among YWSS recruited in two Zimbabwean cities implementing the DREAMS programme than in four towns where DREAMS was not implemented. After adjustment for baseline imbalance in HIV prevalence and predictors of HIV incidence, this potential effect of DREAMS on HIV incidence was not statistically significant. We identified some changes in outcomes on important ‘pathways to impact’: YWSS recruited in DREAMS cities reported fewer clients in the past month, less condom-less sex in the last month and higher levels of PrEP use at 24-month follow-up; yet we found little difference in women’s engagement with social protection services. Despite our null finding, it remains plausible that DREAMS investments may have contributed to reduced HIV incidence among YWSS through increased access to clinical HIV prevention services, including PrEP and condoms.

Our study had limitations, most of which we were aware of from the start.^5^ We considered this study a plausibility evaluation since it was only powered to detect a large difference in HIV incidence and because of the lack of randomisation. A 40% reduction in HIV incidence over 24 months was an ambitious target and full implementation was not achieved until early 2019. Our sample size calculation estimated 70% retention of recruited women, but in practice this was lower, likely affecting the study’s power to detect any difference in HIV incidence as well as potentially introducing bias.^5^ Mobility is high among FSW in Zimbabwe, including YWSS, and during efforts to maintain contact with enrolled women we often heard that they had left the area to seek employment opportunities.

We used data collected by the national FSW programme to identify comparison sites similar to the DREAMS cities based on, among other factors, attendance to the programme and the proportion of attendees aged 18–24.^5^ With the DREAMS cities among the largest and most populous in Zimbabwe, identifying comparison sites was challenging, as evidenced by the higher HIV prevalence in these towns at enrolment. Also, service delivery was less intense and delivered through mobile clinics in all but one non-DREAMS towns, compared with DREAMS cities. The confounding factors, for which we adjusted for in our primary analysis, reduced but did not eliminate differences in HIV prevalence at enrolment. There may, therefore, be other factors that we did not account for that could explain the variation in HIV incidence. Furthermore, although incidence was lower in the two DREAMS cities than in all four non-DREAMS towns, it varied across the few sites included in our study, limiting our ability to draw firm conclusions about any effect of DREAMS on HIV incidence.

Finally, our secondary outcomes were self-reported via face-to-face-interviews and thus potentially prone to social desirability bias and the potential for an ‘intervention’ effect of more frequent contact and follow-ups among the YWSS in DREAMS cities over time.

Intriguingly, in a sensitivity analysis where we dropped seed participants and applied RDS-II weighting, we found a borderline significant impact of the programme aligned with the 40% reduction hypothesised. We decided a priori not to use this weighting in the primary analysis, and consider interpretation of these findings complex, but report them for transparency. At enrolment, RDS diagnostics suggested that women recruited were representative of the network of YWSS in the majority of sites, but convergence was not realised in one of the smaller non-DREAMS towns suggesting that YWSS may not have been representative of the broader network of YWSS in this site.^2^ Furthermore, documenting refusal rates is difficult in RDS design.^13^

Despite these limitations, our study provides invaluable and important evidence in relation to HIV prevention efforts for a critical population. Despite their vulnerability, YWSS are often underrepresented in research.^2 14^ We show that, although challenging, cohort studies with YWSS are feasible. Retention was affected by women’s mobility and likely affected by some women transitioning out of sex work, and therefore no longer interested in participation. Nonetheless, we retained over half the ~2400 women across six sites over the course of the study. Although retention was low compared with a cohort study of young FSW in Burkina Faso, which retained 86% of 321 women recruited over a median of 16.8 months of follow-up,^15^ and a study in Tanzania, which retained 78% of 293 HIV-negative FSW through monthly contacts over 18 months,^16^ there was little evidence of differential follow-up between arms in our study. To retain women, future cohort studies with YWSS should similarly employ a combination of complementary strategies, including use of mobile phone messaging and outreach.

Our findings of a plausible effect of DREAMS, adds an important new finding to a small overall evidence base. In Burkina Faso, a cohort study combined with modelling found a significant effect of integrated prevention and care services combined with peer-led education sessions on HIV incidence among young FSW.^15^ In Benin, a time-series analysis of community-based activities to promote condom use and empower FSW, combined with clinical services and strategies to reduce violence, found a significant impact on HIV prevalence.^17^ In Tanzania, Project Shikamana found that community-led peer education, peer navigation, sensitivity training for HIV care providers and a community-led drop-in centre for activities to promote social cohesion significantly reduced HIV incidence among FSW.^16^ Similar to our study, Project Shikamana included a small number of sites (N=2), limiting their ability to draw inferences of impact at scale.^16^ Unlike most of these studies, our target population included all YWSS in the DREAMS cities, including those who did not identify as FSW, and the intervention included components delivered outside of health facility settings by numerous implementing partners.

Although HIV incidence was lower among YWSS in DREAMS cities, HIV incidence was high in all sites. Our study reiterates that more needs to be done to strengthen implementation of evidence-based HIV prevention interventions for YWSS. YWSS, both those who do and do not self-identify as FSW, are often missed by research and programmes.^2 14 18^ Engaging younger peer outreach workers and specifically targeting younger FSW can increase engagement,^18^ but uptake of programmes tailored to meet the needs of YWSS is often minimal.^19^ Failure to strengthen prevention programming for YWSS, including young FSW, has implications for the health outcomes of women themselves and for broader HIV prevention efforts.

PrEP formed a component of the DREAMS core package. Current use of PrEP among women participating in our study was low at 24 months, consistent with findings reported by PrEP efficacy and implementation studies with AGYW, including FSW, in sub-Saharan Africa,^20 21^ and a number of women in DREAMS cities who initiated PrEP seroconverted. PrEP is an important prevention option for AGYW, providing an opportunity for greater autonomy and control over sexual health.^22^ Recent PrEP studies and our findings underscore the need for approaches to support PrEP initiation and adherence to PrEP during periods of use,^23^ particularly among the many women in our study who reported condom-less sex. Stigma, norms and sexual partners have been shown to influence PrEP use and adherence.^24 25^ Recognising that PrEP and condom use are influenced by women’s broader social environment, HIV prevention programmes need to be holistic in their approach, understanding the reasons underlying poor adherence and providing social support and evidence-based adherence support during periods of PrEP use. Lessons learnt from effective antiretroviral therapy (ART) adherence strategies could inform PrEP adherence support. Wraparound peer-supported community-based treatment improved viral suppression among adolescents aged 13–19 in Zimbabwe,^26^ and long-term virological suppression was achieved among adults attending treatment adherence clubs in South Africa.^27^ With evidence that long-acting PrEP is effective at reducing HIV risk among men who have sex with men and transgender women, ongoing development of alternative formulations of long-acting PrEP holds promise for addressing barriers to oral PrEP for AGYW.^28^ In addition to support for PrEP use, continued condom supply and strengthened distribution alongside strategies to promote condom use, where feasible and when PrEP adherence is low, need continued support and scale-up.^29^

To reduce HIV incidence by 40% over 24 months, DREAMS endeavoured to provide AGYW with a combination of evidence-based HIV prevention interventions to address the multiple factors influencing HIV risk.^30–32^ Termed ‘layering’, the intention was to provide AGYW multiple interventions from the DREAMS core package simultaneously.^30^ Studies show that ‘layering’ was achieved in other DREAMS-supported countries,^31^ yet we found that evidence of successful ‘layering’ of social services alongside comprehensive clinical services for FSW among this particular group of women in Zimbabwe was complex. At follow-up, coverage of clinical services and condoms was generally higher in the DREAMS cities than the four towns. However, coverage of social protection services was low among all women participating in the study. In DREAMS cities, low coverage was driven in part by implementation challenges, including the limited experience of other implementing partners in working with YWSS, and by barriers faced by YWSS in accessing these services. Lessons learnt through the early implementation and delivery of DREAMS to YWSS have been used to streamline service delivery for this group of women. There remains, therefore, scope to evaluate the influence of an adapted form of DREAMS, aimed at being more responsive to the needs of YWSS, on HIV risk among YWSS and learn how best to deliver wraparound social protection services to YWSS.

## Data Availability

Data are available upon request to the London School of Hygiene & Tropical Medicine data repository.
