# Supplementary material for: The impact of the DREAMS partnership on HIV incidence among young women who sell sex in two Zimbabwean cities: results of a non-randomised study
Source: BMJ Glob Health. 2021 Apr 27;6(4):e003892. doi: 10.1136/bmjgh-2020-003892 (PMC8088246; doi:10.1136/bmjgh-2020-003892)
Supplement: Supplementary data [file bmjgh-2020-003892supp001.pdf]

Supplement to: The impact of the DREAMS partnership on HIV incidence among young women who sell sex in two Zimbabwean cities: results of a non-randomised study

APPENDICES

1. Analysis plan
2. Additional descriptive statistics
3. Patterns of retention at 24-month follow-up
4. Comparison of dreams secondary outcomes by site
5. RDS diagnostics and results
6. Sensitivity analysis

APPENDIX 1: ANALYSIS PLAN

A non-randomised evaluation of the impact of the combined DREAMS package of HIV prevention interventions on HIV incidence among young women who sell sex in Zimbabwe

Statistical Analysis Plan

Sungai T. Chabata, CeSHHAR

James R. Hargreaves, Bernadette Hensen, LSHTM; Frances M. Cowan, LSTM and CeSHHAR

Final version 1.9, 20 August 2019

Contents

1. INTRODUCTION 2
2. AIM 2
3. STUDY DESIGN 2
4. SAMPLING 2
5. PRIMARY AND SECONDARY OUTCOMES 3
6. STATISTICAL ANALYSIS: OVERALL APPROACH 6
7. STAGES OF ANALYSIS 9
   i. COHORT RECRUITMENT AND RETENTION 9
   ii. PARTICIPANT CHARACTERISTICS AT ENROLMENT 10
   iii. UPTAKE OF SERVICES 10
   iv. PARICIPANT CHARACTERISTICS AT 24-MONTH FOLLOW UP 10
   v. HIV INCIDENCE RATE BY DISTRICT AND STUDY ARM 10
   vi. UNADJUSTED ANALYSIS 10
   vii. ADJUSTED ANALYSIS 10
   viii. PRE-PLANNED SUB-GROUP ANALYSES 10
   ix. SENSITIVITY ANALYSIS 11
8. STRENGTHS AND LIMITATIONS 11

INTRODUCTION

Worldwide, especially in sub-Saharan Africa, young women are at high risk of HIV due to increased biological, economic and social vulnerability. In Zimbabwe, where the HIV burden is one of the highest in the world and HIV prevalence was 13.3% among adults aged 15-49 years in 2017,[1] adolescent girls and young women (AGYW) aged 15-24 years are at particularly high risk of HIV.

Recognising the vulnerability of AGYW and the social, economic and biological factors that shape women's risk, the DREAMS Partnership was developed to deliver a combined package of interventions targeted at AGYW, their partners, families and communities, to address the interacting factors that shape HIV risk among this particularly vulnerable population. One particular target population for the DREAMS Partnership was young women who sell sex (YWSS). In Zimbabwe, among this group, DREAMS included an offer of oral pre-exposure prophylaxis (PrEP), and condom promotion and provision.

We conducted an impact evaluation of DREAMS among YWSS in Zimbabwe.

AIM

The aim of this study was to estimate the impact of the DREAMS combined package of HIV prevention interventions on HIV incidence among YWSS aged 18-24 years. We also sought to evaluate the impact of DREAMS on a number of secondary outcomes.

STUDY DESIGN

This study was a non-randomised plausibility design to estimate the effect of DREAMS on HIV incidence and other secondary outcomes, highlighted in **section 5**.

We will compare HIV incidence among a cohort of YWSS recruited in two districts where DREAMS was being implemented and followed up over 2 years, to HIV incidence among a cohort of YWSS recruited in four districts where DREAMS was not being implemented and also followed up over 2 years. A similar approach will be used for comparison of secondary outcomes.

SAMPLING

In the two DREAMS sites, a network-based recruitment strategy (respondent-driven sampling (RDS)) was used to identify YWSS in the study communities, offer them HIV testing services and then inform and refer these YWSS to treatment and prevention services, including PrEP and the DREAMS package of HIV prevention interventions through the national programme for sex workers 'Sisters with a Voice' and then onward to the full range of DREAMS services. This process was also used to recruit these women into the evaluation cohort. More specific details of the recruitment process are provided in the protocol (e.g. seed selection, wave recruitment procedures, remuneration).

In two DREAMS sites, eligible YWSS were asked for written informed consent to be interviewed at enrolment into the study in 2017, and then followed-up at 12 and 24 months after the initial enrolment survey. At each time point, rapid HIV testing and counselling was offered to YWSS to ascertain the HIV status of the study participants. A detailed working definition of YWSS is provided in the protocol, but we note here that we included both young women who did and did not self-identify as sex workers, anticipating that the outcome profile might differ among these two groups of young women.

The same network-based recruitment strategy was used to identify and recruit a cohort of YWSS in the four non-DREAMS districts. YWSS recruited in these four districts were also offered HIV counselling and testing services and were referred to the existing national HIV programme for sex

workers, run by the Centre for Sexual Health and HIV/AIDS Research (CeSHHAR). Through this programme they could access HIV prevention services, including condoms, STI treatment and health education, but did not have access to PrEP or other initiatives offered under the DREAMS Partnership. These YWSS were recruited during the same time frame as women in the two DREAMS districts, and were interviewed at enrolment and followed up at 24-months post-enrolment. Although planned, no follow up was done at 12 months among this group due to budgetary constraints.

The difference, therefore, between DREAMS and non-DREAMS sites was that, in DREAMS sites, YWSS had access to comprehensive SRH and HIV services through the Sisters programme PLUS access to PrEP and other DREAMS services, such as social protection interventions. In the non-DREAMS sites, YWSS had access to comprehensive SRH and HIV services through the 'Sisters' programme but were not referred for PrEP / other DREAMS services.

PRIMARY AND SECONDARY OUTCOMES

The primary outcome is incident HIV infection over the 24-month study period, defined as:

Number of new HIV infections among YWSS who tested HIV-negative at enrolment /

Total person-years of follow-up accumulated during the 24-month study period.

Measurement of the primary outcome was restricted to women testing HIV negative at enrolment and followed-up 24-months post-enrolment. HIV status at each round was ascertained through the rapid HIV tests delivered during the counselling and testing process with results returned to participants. No confirmatory testing procedures were conducted for the purpose of the impact evaluation.

Person-years of follow-up was defined as the total follow-up time between the enrolment survey in 2017 and the follow-up survey 24 months after enrolment (2019). For women who seroconverted during the study, the time of seroconversion was set as the mid-point between the enrolment survey and follow-up at 24 months.

The secondary objective of the impact evaluation is to explore whether the DREAMS package of interventions had an impact on the on the secondary outcomes listed in Table 1 and in line with the hypothesised causal pathway through which the DREAMS package of interventions would reduce HIV incidence (Figure 1).

Table 1. Definition of secondary outcomes

Secondary outcome	Definition	Denominator	Numerator
		**Biological protection**	
Knowledge of HIV status	**[Composite outcome] Proportion of YWSS who have EITHER (i) ever tested HIV-positive OR (ii) had an HIV test during the past 6 months and report their HIV-negative result**	All YWSS participating in the survey at 24-month follow-up	YWSS reporting HIV testing in previous 6 months or self-reporting their HIV positive status
Coverage of PrEP	**Proportion of HIV negative YWSS who are currently taking PrEP**	YWSS not testing HIV-positive at 24-month follow up	Number of YWSS self-reporting their HIV-negative status who self-report currently taking PrEP
Uptake of PrEP	**Proportion of HIV negative women who were offered PrEP who accepted the offer**	YWSS self-reporting ever being offered PrEP	Number of YWSS self-reporting that they were ever offered PrEP AND that they accepted the offer of PrEP
Knowledge of partner's status	**Proportion of YWSS who report knowing the HIV status of at least one of their three most recent partners**	All YWSS participating in the 24-month follow up survey	Number of YWSS who report knowing the HIV status of at least one of their three most recent partners
	**Proportion of YWSS who report feeling confident in discussing HIV testing with any partner**	All YWSS participating in the 24-month follow up survey	Number of YWSS who agree/strongly agree that they can discuss HIV testing with any partners
Condom-less sex with regular partner	**Proportion of YWSS who report condom-less sex with regular partner in the past month**	All YWSS participating in the 24-month follow up survey	Number of YWSS reporting any condom less sex with regular partner in the past month
Condom-less sex with client	**Proportion of YWSS who report condom-less sex with client in the past month**	All YWSS participating in the 24-month follow up survey	Number of YWSS reporting any condom less sex with client in the past month
Increased ability to negotiate condom use with any partner	**Proportion of women agreeing that they feel confident in negotiating condom use with any partner**	All YWSS participating in the 24-month follow up survey	Number reporting that they agree/strongly agree that they are confident in negotiating condom use with any sexual partner
Number of sex work clients	**Number of partners with whom women had sex with in exchange for money/material support (i.e. clients) in the past month**	All YWSS participating in the 24-month follow up survey	Categorical variable of <3; 4-9; 9+ clients reported in the past month
Access to STI treatment services	**Proportion of YWSS who self-reported having STI symptoms and seeking of treatment services or advice in the last 12 months**	YWSS reporting having STI symptoms in the past 12 months among YWSS participating in the follow-up survey	Number of YWSS reporting accessing treatment services or advice after having STI symptoms
		**Social Protection**	
Food insecurity	**[Composite outcome] Proportion of YWSS who IN THE PAST 4 WEEKS have EITHER (i) had no food to eat because of lack of resources to get food OR (ii) had a household member who went to bed hungry because there was not enough food OR (iii) had a household member who had a whole day and night without food**	All YWSS participating in the 24-month follow up survey	Number of YWSS responding 'Yes' to either of the three questions on inadequate quantity of food

Reliance on sex work for economic reasons	**Proportion of YWSS who report that they were unable to decline sex with a man because of support offered in the past month**	All YWSS participating in the 24-month follow up survey	Number of YWSS reporting ever unable to decline sex in past month
	**Proportion of YWSS who report that selling sex is the main way they support themselves**	All YWSS participating in the 24-month follow up survey	Number of YWSS reporting that selling sex is the main way they obtain money/support themselves
**Experience of violence**			
Experience of violence from partners	**Proportion of YWSS reporting having had a partner who hit, slapped, kicked, pushed, shoved or otherwise physically hurt her in the previous 12 months**	All YWSS participating in the 24-month follow up survey	Number of YWSS responding that they have been hit, kicked, slapped, shoved by a partner at least once in past 12 months
Experience of violence from police	**Proportion of YWSS reporting that a member of the police hit, slapped, kicked, pushed, shoved or otherwise physically hurt her in the previous 12 months**	All YWSS participating in the 24-month follow up survey	Number of YWSS responding that they have been hit, kicked, slapped, shoved by a member of police at least once in past 12 months

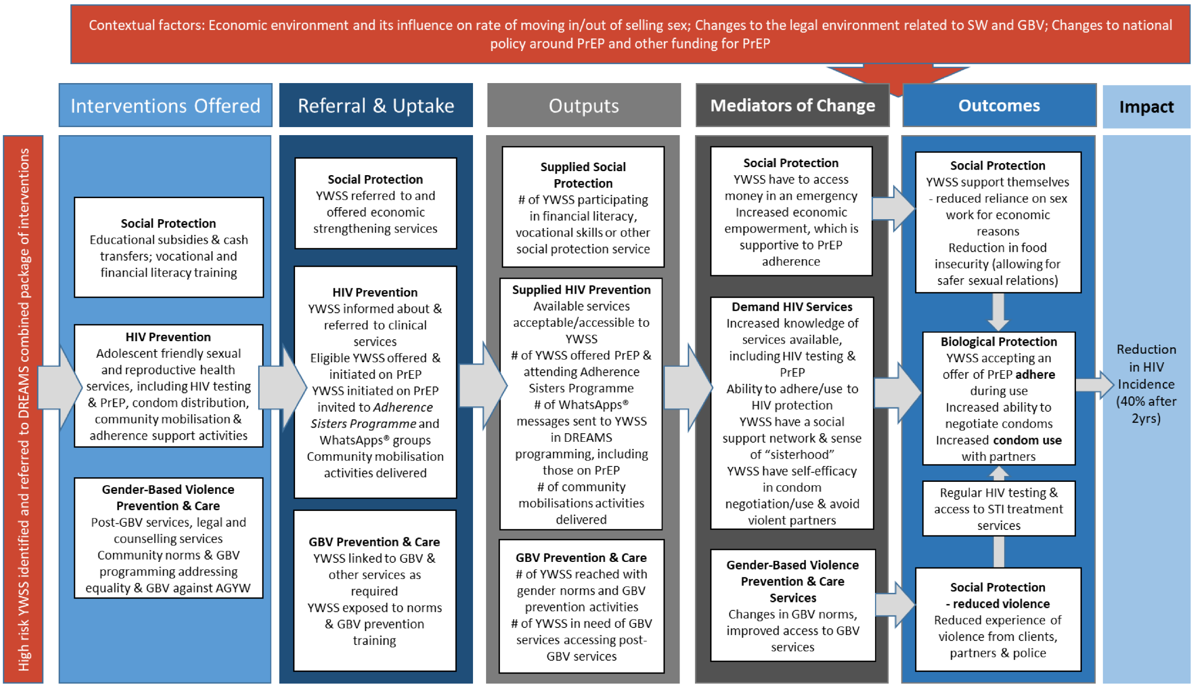

Figure 1. Hypothesised pathway through which the DREAMS combined package of interventions would have an impact on HIV incidence

STATISTICAL ANALYSIS: OVERALL APPROACH

Intention to treat: We will compare the HIV incidence rate between women recruited to the study cohort in the two DREAMS districts to the HIV incidence rate among the women recruited to the study cohort in the four non-DREAMS comparison districts, regardless of individual level of participation in the components of the DREAMS package.

In further analyses, not described here but included as part of the wider impact of evaluation of DREAMS across 4 settings, we plan to explore the relationships between individual level participation in DREAMS interventions and the primary and secondary outcomes in the two districts where DREAMS interventions were delivered.

Individual level analysis: Although the DREAMS package was allocated at district-level, we were not able to include either randomisation or a sufficient number of clusters to undertake a cluster-level randomised trial. Our analysis is therefore based on an individual level analysis comparing the cohorts recruited in the two DREAMS and four non-DREAMS clusters, in which we can evaluate whether outcomes are different across the six clusters but in which the impact of DREAMs cannot be unambiguously distinguished from the "background" differences among the clusters at baseline. Limitations of this "individual-level" analysis approach are described below.

Regression framework: For the primary outcome analysis we will use a Poisson regression framework, appropriate for the rate at which an outcome occurs per unit time. All secondary outcomes are assessed using logistic regression for binary outcomes, measured cross-sectionally after a fixed follow-up period.

Weighting and standard error adjustment for RDS: We recruited participants using RDS. Where RDS is used to make population-level estimates generalizable to the total study population of a particular geography, a variety of statistical procedures and associated assumptions are used to adjust

analyses reflecting this design. In this study the primary aim is to estimate the impact of the DREAMS interventions, by making fair comparisons across the six clusters, and so we will not weight the data to account for recruitment using RDS - on the basis that the approach to sampling was the same/standardised in each cluster. However, we plan a sensitivity analysis in which weighting will be applied; this approach adds an additional layer of complexity, but aims to provide estimates of rates/prevalences for the total study population.

Adjustment for confounding strategy: In the absence of randomisation, and also because the number of study clusters is low, it is appropriate to adjust the analysis for potential confounding variables. If we find evidence for a difference in HIV incidence between the two study arms, caution is needed when considering whether this difference is attributable/partly attributable to DREAMS. We investigated *a priori* what confounding factors to adjust for in order to obtain the fairest comparisons among study clusters. To do this, we used HIV prevalence at baseline as a “proxy” for the background level of HIV incidence.

We found that HIV prevalence was different between the two study arms at baseline. We then identified a set of variables that we *a priori* thought might be associated with HIV prevalence as explored elsewhere,[2] including age at enrolment, highest level of education attained, marital status, self-identification as a sex worker, STI symptoms and number of sexual partners in the past month. We included each variable in a univariable logistic regression model to confirm that they were associated with HIV prevalence and then modelled HIV prevalence against DREAMS arm adjusting for all six variables. Adjusting for these variables attenuated the difference in HIV prevalence between the two arms substantially (see Table 2).

Table 2. Factors associated with HIV prevalence at enrolment

**Characteristic**	**N (%)**	**# of YWSS tested HIV positive at enrolment N=543 n (%)**	**Crude OR (95% CI)**	**P-value**	**Adjusted OR‡ (95% CI)**	**P-value**
**DREAMS**				0.001		0.103
Non-DREAMS sites	1192 (50.2)	312 (25.8)	1		1	
DREAMS sites	1177 (49.8)	231 (19.0)	0.67 (0.53-0.85)		0.81 (0.63-1.04)	
**Age at enrolment**				<0.001		
18-19	814 (36.4)	93 (11.2)	1			
20-24	1555 (63.6)	450 (28.9)	3.20 (2.41-4.26)			
**Highest level of education**				<0.001		
Primary or less	388 (17.0)	135 (34.0)	1			
Incomplete secondary	1050 (44.2)	268 (24.8)	0.64 (0.47-0.86)			
Complete secondary or higher	931 (38.8)	140 (14.7)	0.33 (0.24-0.46)			
**Marital status**				<0.001		
Single/ never married	1385 (60.0)	242 (17.6)	1			
Married/ cohabiting	48 (2.4)	12 (27.2)	1.75 (0.81-3.81)			
Previously married	936 (37.6)	289 (29.9)	2.01 (1.59-2.53)			
**Self-identification as a sex worker**				<0.001		
No	724 (33.1)	113 (15.3)	1			
Yes	1627 (66.9)	429 (26.2)	1.96 (1.50-2.57)			
**STI symptoms**				<0.001		

No	1750 (74.0)	308 (16.9)	1	
Yes	619 (26.0)	235 (38.2)	3.04 (2.39-3.88)	
**Number of sexual partners in the past month**				<0.001
≤1	213 (10.1)	37 (17.8)	1	
2-5	1047 (46.1)	168 (15.7)	0.86 (0.54-1.35)	
6-9	301 (12.3)	66 (20.2)	1.17 (0.70-1.97)	
≥10	808 (31.5)	272 (34.7)	2.46 (1.57-3.86)	

‡Adjusted for DREAMS, age at enrolment, highest level of education attained, marital status, self-identification as FSW, STI symptoms, number of sexual partners in the past month

We will therefore adjust our primary analysis for these six variables, measured at baseline among the cohort, so as to make fairer comparisons between the two DREAMs and four non-DREAMs study clusters, and a fairer attribution of any difference we see to DREAMS intervention. The number of sero-conversions over the two-year study period may be small relative to the number of parameters if all six variables are included in a model. We will, therefore, need to consider the "rule-of-thumb" that the number of sero-conversions should be approximately ten-times higher than the number of parameters included in our model. If the number of sero-conversions is smaller than the number of parameters if all six variables are included, we will prioritise adjusting for the variables that were the strongest confounders of the association between prevalent HIV and DREAMS, including age and educational attainment if there is evidence that the model becomes unstable with the addition of more parameters.

We will not be able to formally adjust our analysis for cluster level factors that may differ between the DREAMS and non-DREAMS districts, for example background HIV prevalence, because we have "only" included six clusters in the study. This is a limitation of our study described in further detail below

At baseline, we also analysed how HIV prevalence increased with age among the YWSS recruited in DREAMS and non-DREAMS districts (see Figure 2). We fitted a simple linear regression line to HIV prevalence by age. We noted that, while there was a difference in the HIV prevalence at each age, as described above, the rate of increase of HIV prevalence with age was almost identical at approximately an average of 6% per single year of age increase in both groups. Among young women, HIV prevalence increasing with age can be cautiously interpreted as reflecting the underlying HIV incidence.

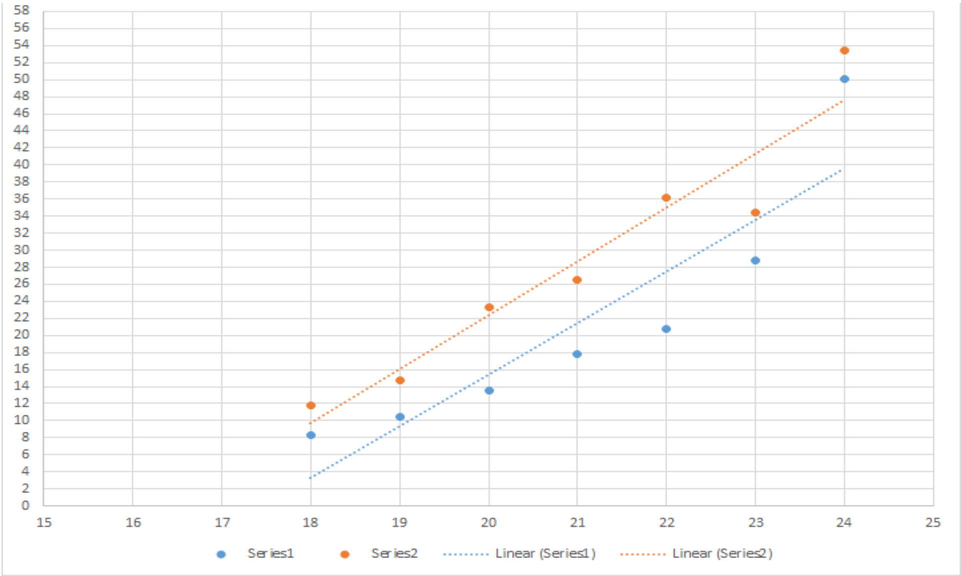

Figure 2: HIV prevalence by age and arm (blue = DREAMS sites and orange = Non-DREAMS sites)

The combination of our data suggesting an

i) HIV prevalence that differed between the arms at baseline, but that could be largely adjusted away through the addition of six key covariates measured at baseline, and which we will adjust our primary outcome analyses for,

and

ii) our finding that at baseline HIV prevalence increasing by age was near identical between the groups,

provide strength to the argument we will make post-analysis, that an adjusted difference in measured HIV incidence between the arms can be plausibly interpreted as reflecting the effect of the DREAMs intervention. The further the adjusted rate ratio is from 0.8, the more credible it is that DREAMS had an effect on HIV incidence. Our findings will be interpreted in the context of DREAMS uptake. We will explore uptake of DREAMS interventions across the two study sites (as described in section iv); where there is evidence of differential uptake across the two groups, this would add credibility to any finding that the DREAMS intervention had an effect on HIV incidence.

Reporting: Analyses will be reported in line with the Transparent Reporting of Evaluations with Nonrandomized Designs (TREND) statement.

STAGES OF ANALYSIS

i. COHORT RECRUITMENT AND RETENTION

We will first describe recruitment into the cohort study, and present a flow chart showing the number of women recruited through the RDS in each district and by study arm (DREAMS and non-DREAMS), and retention at 24-month follow-up.

We will generate RDS recruitment trees by site, colour coding women by whether they tested HIV negative, HIV positive at enrolment, or tested HIV negative at enrolment and seroconverted at 24-month follow up. We have already done the detailed RDS diagnostics which found no evidence of

bias with respect to HIV prevalence in either DREAMS or non-DREAMS sites and reported them elsewhere.[2]

Using data collected on efforts to contact the women at the 24-month follow up, we will describe the reported reasons why women were lost to follow, for example they reportedly migrated and/or married, or were contacted but refused to participate.

ii. PARTICIPANT CHARACTERISTICS AT ENROLMENT

We will describe, by site and study arm, key demographic and behavioural characteristics of the women recruited to the cohort, including those that were identified to be associated with HIV prevalence at enrolment. We will repeat this analysis for YWSS who tested HIV negative at enrolment.

iii. UPTAKE OF SERVICES

The services delivered by DREAMS implementing partners may also be available to the women in non-DREAMS sites, particularly HIV testing service but also educational subsidies, and vocational skills training. Using 24-month follow-up data, we will describe the uptake of other services that may be available in and accessible to women in non-DREAMS sites, by arm and by site.

iv. PARICIPANT CHARACTERISTICS AT 24-MONTH FOLLOW UP

We will describe the proportion of women retained in the study at the 24-month follow-up, and describe follow-up by enrolment characteristics, including: age, marital status, highest level of education attainment and whether women self-identified as a female sex worker.

We will repeat the descriptive analysis presented for enrolment for YWSS testing HIV negative at enrolment and followed up at 24 months.

v. HIV INCIDENCE RATE BY DISTRICT AND STUDY ARM

We will describe the number of new infections observed over the 24-month study period, and describe person-years of follow-up by district and study arm (Table 10). Subsequently, we will estimate the HIV incidence rate among the YWSS by district and by study arm.

vi. UNADJUSTED ANALYSIS

We will use Poisson regression to compare the HIV incidence rates across the two study arms.

We will fit three models, namely:

1) An unadjusted Poisson regression model

vii. ADJUSTED ANALYSIS

We will use Poisson regression models to compare the HIV incidence rates across the two study arms, adjusting for covariates. The models we will fit will be:

2) An age-adjusted Poisson regression model
3) A fully adjusted model including age and individual level potential confounders measured at baseline and found to be associated with HIV prevalence.

viii. PRE-PLANNED SUB-GROUP ANALYSES

We will conduct exploratory analysis, stratifying the analysis by age and by self-identification as a sex worker, to get a sense of difference in the effect size among these subgroups, and will report the findings of these analyses. We however recognise that these analyses will likely be underpowered and should be interpreted cautiously.

ix. SENSITIVITY ANALYSIS

We will perform 2 sets of sensitivity analyses:

1) Our primary analysis strategy does not weight the data. We will exclude seed participants and weight data by the inverse of women's reported YWSS-network size and normalise these weights by site.

2) Our primary analysis excludes data collected from women followed up at 12-months post-enrolment in the DREAMS districts. In our second sensitivity analysis, we will add data from the 12-month survey in the DREAMS districts to obtain information on women not followed up at 24 months. In these DREAMS districts, if someone seroconverted by the time of the 12-month follow-up, we will place the seroconversion date at the mid-point of enrolment and 12 months, and if someone tested HIV-negative at 12 months but HIV-positive at 24 months, we will place the seroconversion at the mid-point 12 months and 24 months. We will then use this information to compare HIV incidence between the two arms.

STRENGTHS AND LIMITATIONS

Our primary analysis is relatively simple, providing descriptions of HIV incidence in each site and unadjusted and adjusted statistical comparisons between the DREAMS and non-DREAMS study arms.

Our adjustment strategy includes a step-wise adjustment process, with few *a priori* variables adjusted for if they were associated with HIV prevalence at enrolment. We therefore consider the analysis to be transparent, and to build an evidence base for whether it is plausible that DREAMS had an impact on HIV incidence.

A limitation of our analysis is that we may not have collected data on important covariates that are associated with HIV and important risk factors for the outcome (HIV incidence), and that differ among study clusters / by arm. As such, if we find evidence for an effect of DREAMS on HIV incidence after adjustment, we cannot be entirely sure that this effect is attributable to DREAMS.

We are unable to conduct cluster level analysis or adjust for cluster level covariates, despite the fact that the intervention was allocated at cluster level. This means we will be unable to adjust for important potential confounders at cluster level; for example, the background HIV prevalence in the DREAMS clusters may differ from that in the non-DREAMS clusters owing to both the lack of randomisation and because of chance variability. Our interpretation will thus need to be cautious, commenting on any differences in cluster level factors that are observed at baseline.

Our intention to treat approach will compare women eligible to receive DREAMS interventions in districts where DREAMS was operating with women in districts where it was not. If DREAMS delivery was weak, or did not reach the specific target populations of women who are the focus for our impact evaluation (YWSS), then we may conclude that DREAMS did not have an impact but this may reflect limited delivery rather than the maximum potential effect. We will describe the delivery and uptake of DREAMS interventions in order to support our interpretation.

APPENDIX 2: ADDITIONAL DESCRIPTIVE STATISTICS

Supplemental Table 1. Key demographic and behavioural characteristics of all YWSS at enrolment by study group, 2017

	DREAMS cities (N=1204) n/N (%)	Non-DREAMS towns (N=1227) n/N (%)	Comparison P-value
**HIV prevalence**	234/1197 (19.5)	320/1216 (26.3)	<0.001
**Age at recruitment**			0.518
18-19	405/1204 (33.6)	428/1227 (34.9)	
20-24	799/1204 (66.4)	799/1227 (65.1)	
**Highest level of education**			<0.001
None/ incomplete primary	41/1204 (3.4)	133/1227 (10.8)	
Complete primary	84/1204 (7.0)	138/1227 (11.3)	
Incomplete secondary	1018/1204 (84.5)	936/1227 (76.3)	
Complete secondary or higher	61/1204 (5.1)	20/1227 (1.6)	
**Marital status**			<0.001
Single/ never married	801/1204 (66.5)	625/1227 (50.9)	
Married / living together as if married	31/1204 (2.6)	19/1227 (1.6)	
Divorced/ separated	365/1204 (30.3)	567/1227 (46.2)	
Widowed	7/1204 (0.6)	16/1227 (1.3)	
**Years selling sex**			0.026
0-2	614/1203 (51.0)	680/1224 (55.6)	
3+	589/1203 (49.0)	544/1224 (44.4)	
**Self-identification as sex worker**			0.031
No	390/1191 (32.7)	350/1220 (28.7)	
Yes	801/1191 (67.3)	870/1220 (71.3)	
**Condom use at last with regular partner**			0.004
No	320/907 (35.3)	384/919 (41.8)	
Yes	587/907 (64.7)	535/919 (58.2)	
**Condom-less sex with regular partner in the past month**			0.479
No	498/908 (54.8)	491/923 (53.2)	
Yes	410/908 (45.2)	432/923 (46.8)	
**Condom use at last with client**			0.302
No	105/946 (11.1)	89/923 (9.6)	
Yes	841/946 (88.9)	834/923 (90.4)	
**Condom-less sex with client in the past month**			0.001
No	786/949 (82.8)	815/924 (88.2)	
Yes	163/949 (17.2)	109/924 (11.8)	
**STI symptoms in the last 12 months**			0.003
No	919/1204 (76.3)	871/1227 (71.0)	
Yes	285/1204 (23.7)	356/1227 (29.0)	
**Risk of common mental disorder**			<0.001
No	733/1204 (60.9)	840/1227 (68.5)	
Yes	471/1204 (39.1)	387/1227 (31.5)	

Chabata ST, *et al. BMJ Global Health* 2022; 6:e003892. doi: 10.1136/bmjgh-2020-003892

Supplemental Table 2. Key demographic and behavioural characteristics of all YWSS at enrolment by study site, 2017

	DREAMS Site A (N=601)	DREAMS Site B (N=603)	Non-DREAMS Site C (N=318)	Non-DREAMS Site D (N=300)	Non-DREAMS Site E (N=308)	Non-DREAMS Site F (N=301)
	n/N (%)	n/N (%)	n/N (%)	n/N (%)	n/N (%)	n/N (%)
**HIV prevalence**	128/597 (21.4)	106/600 (17.7)	84/316 (26.6)	90/299 (30.1)	42/300 (14.0)	104/301 (34.6)
**Age at recruitment**						
18-19	222/601 (36.9)	183/603 (30.3)	103/318 (32.4)	101/300 (33.7)	142/308 (46.1)	82/301 (27.2)
20-24	379/601 (63.1)	420/603 (69.7)	215/318 (67.6)	199/300 (66.3)	166/308 (53.9)	219/301 (72.8)
**Highest level of education**						
None/ incomplete primary	12/601 (2.0)	29/603 (4.8)	56/318 (17.6)	34/300 (11.3)	13/308 (4.2)	30/301 (10.0)
Complete primary	49/601 (8.2)	35/603 (5.8)	40/318 (12.6)	37/300 (12.3)	21/308 (6.8)	40/301 (13.3)
Incomplete secondary	512/601 (85.2)	506/603 (83.9)	219/318 (68.9)	227/300 (75.7)	271/308 (88.0)	219/301 (72.8)
Complete secondary or higher	28/601 (4.7)	33/603 (5.5)	3/318 (0.9)	2/300 (0.7)	3/308 (1.0)	12/301 (4.0)
**Marital status**						
Single/ never married	501/601 (83.4)	300/603 (49.8)	135/318 (42.5)	130/300 (43.3)	197/308 (64.0)	163/301 (54.2)
Married / living together as if married	22/601 (3.7)	9/603 (1.5)	2/318 (0.6)	3/300 (1.0)	12/308 (3.9)	2/301 (0.7)
Divorced/ separated	75/601 (12.5)	290/603 (48.1)	172/318 (54.1)	164/300 (54.7)	99/308 (32.1)	132/301 (43.9)
Widowed	3/601 (0.5)	4/603 (0.7)	9/318 (2.8)	3/300 (1.0)	0/308 (0.0)	4/301 (1.3)
**Years selling sex**						
0-2	291/600 (48.5)	323/603 (53.6)	180/318 (56.6)	173/298 (58.1)	167/307 (54.4)	160/301 (53.2)
3+	309/600 (51.5)	280/603 (46.4)	138/318 (43.4)	125/298 (41.9)	140/307 (45.6)	141/301 (46.8)
**Self-identification as sex worker**						
No	223/590 (37.8)	167/601 (27.8)	65/313 (20.8)	69/300 (23.0)	151/306 (49.3)	65/301 (21.6)
Yes	367/590 (62.2)	434/601 (72.2)	248/313 (79.2)	231/300 (77.0)	155/306 (50.7)	236/301 (78.4)
**Condom use at last with regular partner**						
No	201/476 (42.2)	119/431 (27.6)	84/227 (37.0)	93/235 (39.6)	127/278 (45.7)	80/179 (44.7)
Yes	275/476 (57.8)	312/431 (72.4)	143/227 (63.0)	142/235 (60.4)	151/278 (54.3)	99/179 (55.3)
**Condom-less sex with regular partner in the past month**						
No	231/477 (48.4)	267/431 (61.9)	126/228 (55.3)	116/238 (48.7)	144/278 (51.8)	105/179 (58.7)
Yes	246/477 (51.6)	164/431 (38.1)	102/228 (44.7)	122/238 (51.3)	134/278 (48.2)	74/179 (41.3)
**Condom use at last with client**						
No	71/490 (14.5)	34/456 (7.5)	16/255 (6.3)	19/234 (8.1)	30/191 (15.7)	24/243 (9.9)
Yes	419/490 (85.5)	422/456 (92.5)	239/255 (93.7)	215/234 (91.9)	161/191 (84.3)	219/243 (90.1)
**Condom-less sex with client in the past month**						
No	393/491 (80.0)	393/458 (85.8)	236/255 (92.5)	216/234 (92.3)	151/192 (78.6)	212/243 (87.2)
Yes	98/491 (20.0)	65/458 (14.2)	19/255 (7.5)	18/234 (7.7)	41/192 (21.4)	31/243 (12.8)
**STI symptoms in the last 12 months**						

No	470/601 (78.2)	449/603 (74.5)	235/318 (73.9)	185/300 (61.7)	248/308 (80.5)	203/301 (67.4)
Yes	131/601 (21.8)	154/603 (25.5)	83/318 (26.1)	115/300 (38.3)	60/308 (19.5)	98/301 (32.6)
**Risk of common mental disorder**						
No	366/601 (60.9)	367/603 (60.9)	205/318 (64.5)	189/300 (63.0)	218/308 (70.8)	228/301 (75.7)
Yes	235/601 (39.1)	236/603 (39.1)	113/318 (35.5)	111/300 (37.0)	90/308 (29.2)	73/301 (24.3)

Supplemental Table 3. Key demographic and behavioural characteristics at enrolment of YWSS testing HIV negative and followed up at 24 months by group, 2019

	DREAMS cities (N=538) n/N (%)	Non-DREAMS towns (N=481) n/N (%)	Comparison P-value
**Age at enrolment**			0.948
18-19	208/538 (38.7)	185/481 (38.5)	
20-24	330/538 (61.3)	296/481 (61.5)	
**Highest level of education**			<0.001
None/ incomplete primary	11/538 (2.0)	24/480 (5.0)	
Complete primary	27/538 (5.0)	61/480 (12.7)	
Incomplete secondary	449/538 (83.5)	380/480 (79.2)	
Complete secondary or higher	51/538 (9.5)	15/480 (3.1)	
**Marital status**			<0.001
Single/ never married	308/538 (57.2)	182/480 (37.9)	
Married / living together as if married	78/538 (14.5)	58/480 (12.1)	
Divorced/ separated	152/538 (28.3)	234/480 (48.8)	
Widowed	0/538 (0.0)	6/480 (1.3)	
**Years selling sex**			<0.001
0-2	67/537 (12.5)	99/468 (21.2)	
3+	470/537 (87.5)	369/468 (78.8)	
**Self-identification as sex worker**			0.005
No	217/536 (40.5)	154/480 (32.1)	
Yes	319/536 (59.5)	326/480 (67.9)	
**Condom use at last with regular partner**			0.009
No	170/421 (40.4)	182/367 (49.6)	
Yes	251/421 (59.6)	185/367 (50.4)	
**Condom-less sex with regular partner in the past month**			0.004
No	173/445 (38.9)	124/418 (29.7)	
Yes	272/445 (61.1)	294/418 (70.3)	
**Condom use at last with client**			0.153
No	38/394 (9.6)	44/339 (13.0)	
Yes	356/394 (90.4)	295/339 (87.0)	
**Condom-less sex with client in the past month**			0.029
No	321/398 (80.7)	262/354 (74.0)	
Yes	77/398 (19.3)	92/354 (26.0)	
**STI symptoms in the last 12 months**			0.015
No	464/538 (86.2)	386/479 (80.6)	
Yes	74/538 (13.8)	93/479 (19.4)	
**Risk of common mental disorder**			0.450
No	388/538 (72.1)	357/481 (74.2)	
Yes	150/538 (27.9)	124/481 (25.8)	

APPENDIX 3: PATTERNS OF RETENTION AT 24-MONTH FOLLOW-UP

Supplemental Table 4. Age, marital status, whether women self-identified as a female sex worker and educational attainment at enrolment among women testing HIV negative at enrolment and retained or not retained at 24-month follow-up in 2019

	DREAMS cities (N=963)			Non-DREAMS towns (N=896)		
**Follow-up rate**	538/963 (55.9%)			481/896 (53.7%)		
	**YWSS retained (N=538)** n/N (%)	**YWSS not retained (N=425)** n/N (%)	**P-value‡**	**YWSS retained (N=481)** n/N (%)	**YWSS not retained (N=415)** n/N (%)	**P-value‡**
**Age at enrolment**			0.397			0.046
18-19	208/538 (38.7)	153/425 (36.0)		185/481 (38.5)	187/415 (45.1)	
20-24	330/538 (61.3)	272/425 (64.0)		296/481 (61.5)	228/415 (54.9)	
**Marital status**			0.221			0.302
Single/never married	361/538 (67.1)	307/425 (72.2)		256/481 (53.2)	241/415 (58.1)	
Married/cohabiting	12/538 (2.2)	9/425 (2.1)		8/481 (1.7)	8/415 (1.9)	
Previously married	165/538 (30.7)	109/425 (25.6)		217/481 (45.1)	166/415 (40.0)	
**Whether self-identifies as FSW**			0.119			0.582
No	167/532 (31.4)	152/420 (36.2)		159/480 (33.1)	143/410 (34.9)	
Yes	365/532 (68.6)	268/420 (63.8)		321/480 (66.9)	267/410 (65.1)	
**Educational attainment**			0.927			0.163
None/ incomplete primary	15/538 (2.8)	13/425 (3.1)		34/481 (7.1)	43/415 (10.4)	
Complete primary	32/538 (5.9)	29/425 (6.8)		53/481 (11.0)	39/415 (9.4)	
Incomplete secondary	460/538 (85.5)	357/425 (84.0)		386/481 (80.2)	321/415 (77.3)	
Complete secondary or higher	31/538 (5.8)	26/425 (6.1)		8/481 (1.7)	12/415 (2.9)	

‡Chi-square P value for the association of each characteristic with retention at 24-month follow-up

APPENDIX 4: COMPARISON OF DREAMS SECONDARY OUTCOMES BY SITE

Supplemental Table 5. Comparison of DREAMS secondary outcomes by site

	DREAMS Site A (N=252) n/N (%)	DREAMS Site B (N=286) n/N (%)	Non-DREAMS Site C (N=122) n/N (%)	Non-DREAMS Site D (N=102) n/N (%)	Non-DREAMS Site E (N=141) n/N (%)	Non-DREAMS Site F (N=115) n/N (%)
**Improved access to clinical services and HIV prevention services**						
**Knowledge of HIV status**						
No	71/252 (28.2)	48/286 (16.8)	27/122 (22.1)	19/102 (18.6)	37/141 (26.3)	34/115 (29.6)
Yes	181/252 (71.8)	238/286 (83.2)	95/122 (77.9)	83/102 (81.4)	104/141 (73.7)	81/115 (70.4)
Unadjusted	1	1.94 (1.28-2.94), p=0.002	1.38 (0.83-2.29), p=0.214	1.71 (0.97-3.03), p=0.064	1.07 (0.68-1.70), p=0.763	0.93 (0.58-1.52), p=0.785
Fully-adjusted‡	1	1.77 (1.14-2.74), p=0.012	1.25 (0.72-2.15), p=0.432	1.50 (0.82-2.76), p=0.186	1.03 (0.64-1.68), p=0.893	0.83 (0.50-1.38), p=0.473
**Ever taken PrEP**						
No	200/252 (79.4)	187/286 (65.4)	121/122 (99.2)	102/102 (100.0)	141/142 (99.3)	114/115 (99.1)
Yes	52/252 (20.6)	99/286 (34.6)	1/122 (0.8)	0/102 (0.0)	1/142 (0.7)	1/115 (0.9)
Unadjusted	1	2.04 (1.38-3.01), p<0.001	–	–	–	–
Fully-adjusted‡	1	1.83 (1.16-2.91), p=0.010	–	–	–	–
**Ability to negotiate condom use with any partner**						
No	26/252 (10.3)	14/286 (4.9)	5/122 (4.1)	6/102 (5.9)	55/141 (39.0)	25/115 (21.7)
Yes	226/252 (89.7)	272/286 (95.1)	117/122 (95.9)	96/102 (94.1)	86/141 (61.0)	90/115 (78.3)
Unadjusted	1	2.24 (1.14-4.38), p=0.019	2.69 (1.01-7.19), p=0.048	1.84 (0.73-4.62), p=0.193	0.18 (0.11-0.31), p<0.001	0.41 (0.23-0.76), p=0.004
Fully-adjusted‡	1	2.06 (1.02-4.18), p=0.044	2.19 (0.79-6.04), p=0.132	1.47 (0.57-3.83), p=0.428	0.16 (0.09-0.28), p<0.001	0.39 (0.21-0.73), p=0.003
**Knowledge of the HIV status of at least one of their three most recent partners**						
No	68/250 (27.2)	111/278 (39.9)	45/121 (37.2)	32/100 (32.0)	65/139 (46.8)	68/114 (59.6)
Yes	182/250 (72.8)	167/278 (60.1)	76/121 (62.8)	68/100 (68.0)	74/139 (53.2)	46/114 (40.4)
Unadjusted	1	0.56 (0.39-0.81), p=0.002	0.63 (0.40-1.01), p=0.051	0.79 (0.48-1.31), p=0.370	0.43 (0.28-0.66), p<0.001	0.25 (0.16-0.40), p<0.001
Fully-adjusted‡	1	0.64 (0.43-0.96), p=0.030	0.85 (0.52-1.41), p=0.541	1.02 (0.59-1.74), p=0.953	0.44 (0.28-0.70), p<0.001	0.32 (0.19-0.52), p<0.001
**Condom-less sex with regular partner in the past month**						

No	122/251 (48.6)	153/285 (53.7)	58/122 (47.5)	41/100 (41.0)	52/141 (36.9)	48/115 (41.7)
Yes	129/251 (51.4)	132/285 (46.3)	64/122 (52.5)	59/100 (59.0)	89/141 (63.1)	67/115 (58.3)
Unadjusted	1	0.82 (0.58-1.15), p=0.241	1.04 (0.68-1.61), p=0.847	1.36 (0.85-2.18), p=0.198	1.62 (1.06-2.47), p=0.025	1.32 (0.85-2.06), p=0.222
Fully-adjusted‡	1	1.12 (0.73-1.71), p=0.606	1.37 (0.79-2.39), p=0.263	1.31 (0.74-2.31), p=0.358	1.58 (0.98-2.55), p=0.059	1.58 (0.89-2.81), p=0.119
**Condom-less sex with client in the past month**						
No	214/250 (85.6)	261/285 (91.6)	109/122 (89.3)	92/100 (92.0)	106/141 (75.2)	89/115 (77.4)
Yes	36/250 (14.4)	24/285 (8.4)	13/122 (10.7)	8/100 (8.0)	35/141 (24.8)	26/115 (22.6)
Unadjusted	1	0.55 (0.32-0.94), p=0.031	0.71 (0.36-1.39), p=0.318	0.52 (0.23-1.16), p=0.108	1.96 (1.17-3.30), p=0.011	1.74 (0.99-3.05), p=0.054
Fully-adjusted‡	1	0.61 (0.32-1.18), p=0.144	0.60 (0.26-1.42), p=0.247	0.62 (0.25-1.57), p=0.317	2.30 (1.21-4.37), p=0.011	1.98 (1.01-3.86), p=0.045
**Accessed STI treatment services in the past 12 months**						
No	2/32 (6.3)	5/42 (11.9)	3/20 (15.0)	2/32 (6.3)	8/21 (38.1)	5/20 (25.0)
Yes	30/32 (93.8)	37/42 (88.1)	17/20 (85.0)	30/32 (93.8)	13/21 (61.9)	15/20 (75.0)
Unadjusted	1	0.49 (0.09-2.72), p=0.418	0.38 (0.06-2.49), p=0.312	–	0.11 (0.02-0.58), p=0.010	0.20 (0.03-1.15), p=0.072
Fully-adjusted‡	1	–	0.22 (0.01-12.18), p=0.463	–	0.25 (0.01-5.52), p=0.383	–
**Improved coverage of Social and Economic Protection service**						
**Food insecurity**						
No	191/252 (75.8)	176/286 (61.5)	35/122 (28.7)	54/102 (52.9)	118/140 (84.3)	88/115 (76.5)
Yes	61/252 (24.2)	110/286 (38.5)	87/122 (71.3)	48/102 (47.1)	22/140 (15.7)	27/115 (23.5)
Unadjusted	1	1.96 (1.35-2.84), p<0.001	7.78 (4.78-12.66), p<0.001	2.78 (1.72-4.52), p<0.001	0.58 (0.34-1.01), p=0.050	0.96 (0.57-1.61), p=0.880
Fully-adjusted‡	1	1.56 (1.04-2.35), p=0.034	5.66 (3.34-9.61), p<0.001	2.52 (1.48-4.29), p=0.001	0.54 (0.31-0.95), p=0.033	0.87 (0.51-1.51), p=0.626
**Selling sex is the main way to support myself**						
No	105/252 (41.7)	91/286 (31.8)	37/122 (30.3)	34/102 (33.3)	60/140 (42.9)	28/115 (24.3)
Yes	147/252 (58.3)	195/286 (68.2)	85/122 (69.7)	68/102 (66.7)	80/140 (57.1)	87/115 (75.7)
Unadjusted	1	1.53 (1.08-2.18), p=0.018	1.64 (1.04-2.60), p=0.035	1.43 (0.88-2.31), p=0.147	0.95 (0.63-1.45), p=0.819	2.22 (1.35-3.64), p=0.002
Fully-adjusted‡	1	1.32 (0.90-1.94), p=0.150	1.18 (0.72-1.94), p=0.507	1.13 (0.67-1.89), p=0.655	0.97 (0.63-1.51), p=0.906	2.09 (1.25-3.49), p=0.005
**Ever been unable to decline sex in the past month**						
Never/not in the past month	129/252 (51.2)	128/282 (45.4)	64/119 (53.8)	53/99 (53.5)	76/141 (53.9)	43/115 (37.4)
At least once in the past month	123/252 (48.8)	154/282 (54.6)	55/119 (46.2)	46/99 (46.5)	65/141 (46.1)	72/115 (62.6)

Unadjusted	1	1.26 (0.90-1.77), p=0.181	0.90 (0.58-1.40), p=0.641	0.91 (0.57-1.45), p=0.692	0.90 (0.59-1.36), p=0.606	1.76 (1.12-2.76), p=0.014
Fully-adjusted‡	1	1.25 (0.87-1.80), p=0.235	0.89 (0.55-1.44), p=0.633	0.89 (0.54-1.47), p=0.649	0.95 (0.62-1.45), p=0.805	1.72 (1.08-2.74), p=0.023
**Number of sex work clients in the past month**						
≤3	138/252 (54.8)	145/285 (50.9)	39/116 (33.6)	38/96 (39.6)	86/141 (61.0)	40/115 (34.8)
>3	114/252 (45.2)	140/285 (49.1)	77/116 (66.4)	58/96 (60.4)	55/141 (39.0)	75/115 (65.2)
Unadjusted	1	1.17 (0.83-1.64), p=0.368	2.39 (1.51-3.78), p<0.001	1.85 (1.15-2.98), p=0.012	0.77 (0.51-1.18), p=0.232	0.27 (1.44-3.58), p<0.001
Fully-adjusted‡	1	1.12 (0.76-1.63), p=0.574	1.87 (1.13-3.10), p=0.015	1.56 (0.92-2.64), p=0.100	1.10 (0.70-1.74), p=0.671	2.28 (1.38-3.75), p=0.001
**Gender-based violence prevention, and care and support services**						
**Experience of violence from partners in the past 12 months**						
No	210/252 (83.3)	224/286 (78.3)	76/122 (62.3)	73/102 (71.6)	108/142 (76.1)	88/115 (76.5)
Yes	42/252 (16.7)	62/286 (21.7)	46/122 (37.7)	29/102 (28.4)	34/142 (23.9)	27/115 (23.5)
Unadjusted	1	1.38 (0.90-2.14), p=0.143	3.03 (1.85-4.96), p<0.001	1.99 (1.15-3.42), p=0.013	1.57 (0.95-2.62), p=0.080	1.53 (0.89-2.64), p=0.123
Fully-adjusted‡	1	1.14 (0.71-1.83), p=0.583	2.30 (1.34-3.96), p=0.003	1.39 (0.77-2.49), p=0.275	1.56 (0.91-2.66), p=0.104	1.56 (0.88-2.76), p=0.129
**Experience of violence from police in the past 12 months**						
No	249/252 (98.8)	282/286 (98.6)	122/122 (100.0)	102/102 (100.0)	137/140 (97.9)	112/115 (97.4)
Yes	3/252 (1.2)	4/286 (1.4)	0/122 (0.0)	0/102 (0.0)	3/140 (2.1)	3/115 (2.6)
Unadjusted	1	1.18 (0.26-5.31), p=0.832	–	–	1.82 (0.36-9.13), p=0.468	2.22 (0.44-11.19), p=0.332
Fully-adjusted‡	1	0.87 (0.17-4.44), p=0.869	–	–	1.81 (0.33-9.74), p=0.492	1.62 (0.29-8.95), p=0.581

‡Adjusted for age, highest level of education attained, marital status, self-identification as FSW (measured at baseline), and for each respective secondary outcome measured at baseline

APPENDIX 5: RDS DIAGNOSTICS AND RESULTS

RDS diagnostics were based on enrolment (2017) data. Combined convergence and bottleneck plots (reported elsewhere),[2] suggested that key characteristics and outcomes, including age, whether women self-identified as FSW and HIV prevalence, stabilised with increasing sample sizes in five of the six sites. There was also little evidence of disconnected networks of YWSS in each site. In this study, we report the proportion of women who said that they were recruited into the study by strangers, to have an understanding of reciprocity, and assessed recruitment homophily with respect to HIV status, to understand if HIV positive recruiters preferentially recruited HIV positive peers from amongst their personal networks.

We generated RDS recruitment trees by site, colour coding women by whether they tested HIV negative at enrolment, HIV positive at enrolment, or tested HIV negative at enrolment and seroconverted at 24-month follow up. Detailed RDS diagnostics for these data are presented elsewhere.[2]

Supplemental Table 6. Proportion recruited by strangers, and recruitment homophily for HIV status

Site	Proportion recruited by strangers	Homophily for HIV status
DREAMS Site A	7.1	1.0
DREAMS Site B	21.1	1.0
Non-DREAMS Site C	5.7	1.0
Non-DREAMS Site D	20.6	0.9
Non-DREAMS Site E	21.8	1.0
Non-DREAMS Site F	27.8	1.0

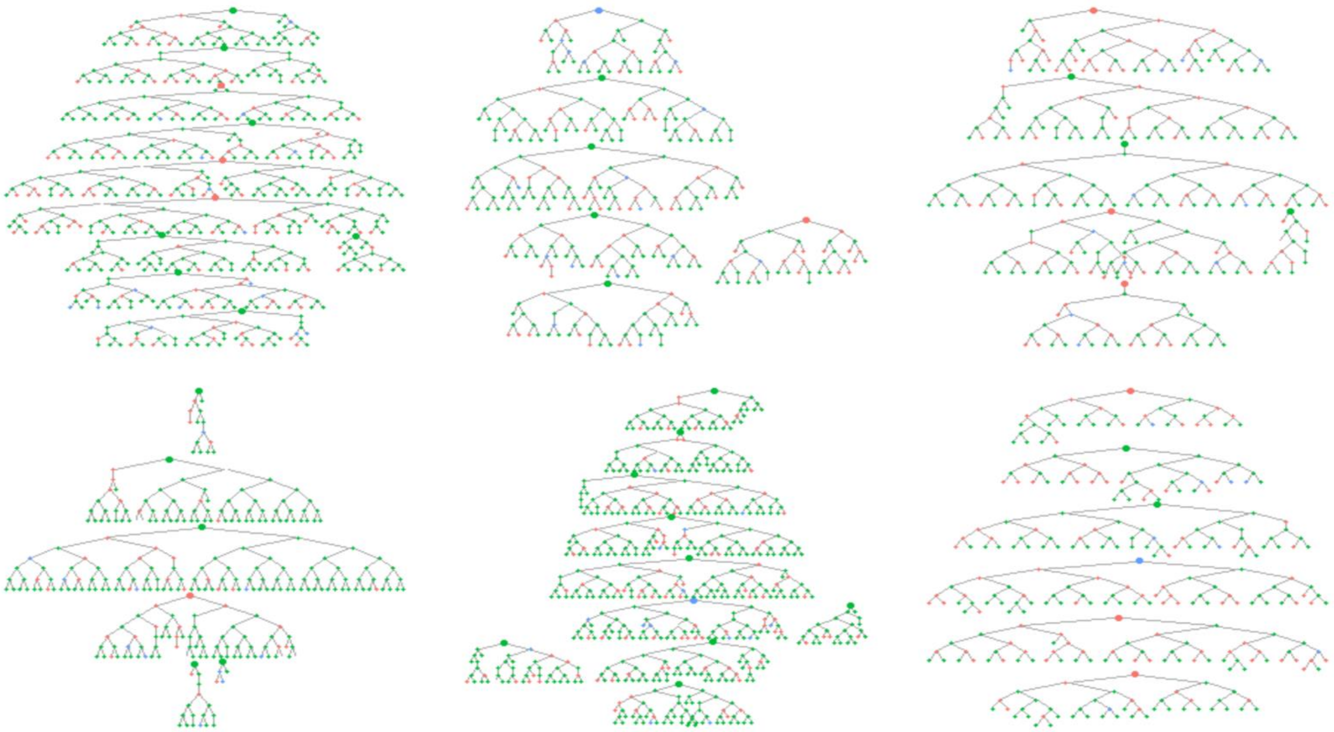

Supplemental Figure 1. Recruitment trees. Red circles represent women who tested HIV positive at enrolment, green circles represent women who tested HIV negative at enrolment, and blue circles represent women who tested HIV negative at enrolment who seroconverted at 24-month follow-up. The larger circles denote speed participants.

Chabata ST, *et al. BMJ Global Health* 2022; 6:e003892. doi: 10.1136/bmjgh-2020-003892

Supplemental Table 7. Follow-up interview questions to assess whether respondent-driven sampling strategy worked well in 2017

		DREAMS Site A‡ (N=137) n/N (%)	DREAMS Site B (N=270) n/N (%)	Non-DREAMS Site C (N=120) n/N (%)	Non-DREAMS Site D (N=96) n/N (%)	Non-DREAMS Site E (N=153) n/N (%)	Non-DREAMS Site F (N=120) n/N (%)
**Apart from the woman who did recruit you to the study, has anyone else approached you to give you a coupon?**							
	No	38/45 (84.4)	248/270 (91.9)	88/120 (73.3)	70/96 (72.9)	151/153 (98.7)	119/120 (99.2)
	Yes	7/45 (15.6)	22/270 (8.1)	32/120 (26.7)	26/96 (27.1)	2/153 (1.3)	1/120 (0.8)
**If yes, how many times has this happened?**							
	Once	1/5 (20.0)	16/22 (72.7)	11/32 (34.4)	16/24 (66.7)	–	–
	Twice	3/5 (60.0)	5/22 (22.7)	12/32 (37.5)	6/24 (25.0)	–	–
	Three or more times	1/5 (20.0)	1/22 (4.5)	9/32 (28.1)	2/24 (8.3)	–	–
**How many YWSS (aged over 18) do you know personally who live in this site, where you know their name and they know yours**							
	median (p25-p75)	6 (5-10)	5 (4-10)	10 (5-15)	6 (4-10)	6 (5-15)	6 (4-10)
**Of these YWSS whom you know personally, how many have you seen in the last month?**							
	median (p25-p75)	5 (4-9)	4 (3-7)	8 (5-10)	6 (4-10)	5 (3-10)	5 (4-10)
**Of the YWSS whom you know personally, how many would you have considered asking to take part in the study?**							
	median (p25-p75)	5 (4-8)	4 (2-7)	4 (2-7)	4 (2-7)	5 (2-10)	4 (2-7)
**How many women did you give a coupon to who accepted it?**							
	0	0/44 (0.0)	2/270 (0.7)	1/117 (0.9)	0/95 (0.0)	0/153 (0.0)	0/120 (0.0)
	1	2/44 (4.5)	5/270 (1.9)	11/117 (9.4)	7/95 (7.4)	19/153 (12.4)	12/120 (10.0)
	2	42/44 (95.5)	263/270 (97.4)	105/117 (89.7)	88/95 (92.6)	134/153 (87.6)	108/120 (90.0)
**How many women did you try to recruit to the survey but who refused?**							
	0	37/44 (84.1)	226/256 (88.3)	88/118 (74.6)	68/94 (72.3)	133/153 (86.9)	106/117 (90.6)
	1	2/44 (4.5)	12/256 (4.7)	16/118 (13.6)	11/94 (11.7)	14/153 (9.2)	9/117 (7.7)
	2	3/44 (6.8)	10/256 (3.9)	8/118 (6.8)	11/94 (11.7)	2/153 (1.3)	0/117 (0.0)
	≥3	2/44 (4.5)	8/256 (3.1)	6/118 (5.1)	4/94 (4.3)	4/153 (2.6)	2/117 (1.7)

Chabata ST, *et al. BMJ Global Health* 2022; 6:e003892. doi: 10.1136/bmjgh-2020-003892

‡Lot of missing data in site A due to errors in questionnaire skip patterns

Across all the study sites, some women were approached more than once to participate in the study (Supplemental Table 7), and very few women refused the coupon they were offered.

APPENDIX 6: SENSITIVITY ANALYSES

We conducted sensitivity analyses in which we (i) included data collected from women followed up at 12-months post-enrolment in the DREAMS sites, and (ii) RDS-II weighted our data, where the 24-momth data was weighted using RDS weights that were generated using enrolment. For (i), we only present the primary outcome results which are very similar to that of the primary analysis results, and we do not present the results of uptake of DREAMS and secondary outcomes results because they are also very similar to that of primary analysis.

Supplemental Table 8. HIV incidence among YWSS testing HIV negative at enrolment, by arm (A) and site (B) (N=1138) (including 12-month follow-up data)

**A. Comparison of HIV incidence among YWSS testing HIV negative at enrolment, by group**				
	**Number of seroconversions/person-years of follow-up**	**Rate per 100 person-years (95% CI)**	**Age-adjusted rate ratio (95%CI) p-value**	**Fully adjusted rate ratio (95%CI)‡ p-value**
**Non-DREAMS (N=479)**	48/907.62	5.29 (3.99-7.02)	1.0	1.0
**DREAMS (N=659)**	42/1098.31	3.82 (2.83-5.17)	0.72 (0.47-1.09) p=0.118	0.79 (0.47-1.32) p=0.364
**B. Comparison of HIV incidence among YWSS testing HIV negative at enrolment, by site**				
**DREAMS Site A (n=309)**	24/493.94	4.86 (3.26-7.25)	1.0	
**DREAMS Site B (n=350)**	18/604.38	2.98 (1.88-4.73)	0.60 (0.32-1.11) p=0.101	0.61 (0.32-1.17) p=0.139
**Non-DREAMS Site C (n=121)**	16/226.24	7.07 (4.33-11.54)	1.44 (0.76-2.71) p=0.263	1.29 (0.64-2.59) p=0.471
**Non-DREAMS Site D (n=102)**	11/192.90	5.70 (3.16-10.30)	1.16 (0.57-2.38) p=0.678	1.12 (0.51-2.42) p=0.783
**Non-DREAMS Site E (n=141)**	12/278.41	4.31 (2.45-7.59)	0.90 (0.45-1.80) p=0.761	0.88 (0.41-1.90) p=0.743
**Non-DREAMS Site F (n=115)**	9/210.07	4.28 (2.23-8.23)	0.86 (0.40-1.86) p=0.705	0.81 (0.36-1.80) p=0.604

‡Adjusted for age, highest level of education attained, marital status, self-identification as FSW, STI symptoms, number of sexual partners in the past month, HIV prevalence (measured at enrolment)

Supplemental Table 9. HIV incidence among YWSS testing HIV negative at enrolment, by group (A) and site (B) (N=1017) (RDS weighted)

	Number of seroconversions/person-years of follow-up	Rate per 100 person-years (95% CI)	Age-adjusted rate ratio (95%CI) p-value	Fully adjusted rate ratio (95%CI)‡ p-value
**A. Comparison of HIV incidence among YWSS testing HIV negative at enrolment, by group**				
**Non-DREAMS (N=479)**	48/907.62	5.61 (3.77-7.46)	1.0	1.0
**DREAMS (N=538)**	31/988.14	2.93 (1.68-4.18)	0.52 (0.31-0.90) p=0.019	0.55 (0.28-1.08) p=0.082
**B. Comparison of HIV incidence among YWSS testing HIV negative at enrolment, by site**				
**DREAMS Site A (n=252)**	16/444.74	3.49 (1.44-5.53)	1.0	
**DREAMS Site B (n=286)**	15/543.40	2.46 (0.92-4.01)	0.69 (0.28-1.66) p=0.401	0.67 (0.28-1.58) p=0.357
**Non-DREAMS Site C (n=121)**	16/226.24	6.63 (2.60-10.66)	1.85 (0.79-4.28) p=0.154	1.47 (0.61-3.55) p=0.395
**Non-DREAMS Site D (n=102)**	11/192.90	6.65 (2.21-11.10)	1.85 (0.76-4.51) p=0.174	1.55 (0.58-4.12) p=0.383
**Non-DREAMS Site E (n=141)**	12/278.41	5.36 (1.94-8.77)	1.54 (0.65-3.66) p=0.328	1.51 (0.60-3.77) p=0.379
**Non-DREAMS Site F (n=115)**	9/210.07	3.88 (1.13-6.63)	1.07 (0.42-2.71) p=0.885	1.10 (0.42-2.85) p=0.849

‡Adjusted for age, highest level of education attained, marital status, self-identification as FSW, STI symptoms, number of sexual partners in the past month, HIV prevalence (measured at enrolment)

Chabata ST, *et al. BMJ Global Health* 2022; 6:e003892. doi: 10.1136/bmjgh-2020-003892

Supplemental Table 10. HIV incidence among young women who sell sex testing HIV negative at enrolment, by arm (A) and site (B) (N=1017) (Imputed seroconversion date)

A. Comparison of HIV incidence among YWSS testing HIV negative at enrolment, by arm				
	**Number of seroconversions/person-years of follow-up**	**Rate per 100 person-years (95% CI)**	**Age-adjusted rate ratio (95%CI) p-value**	**Fully adjusted rate ratio (95%CI)‡ p-value**
**Non-DREAMS (N=479)**	48/907.60	5.29 (3.99-7.02)	1.0	1.0
**DREAMS (N=538)**	31/983.77	3.15 (2.22-4.48)	0.59 (0.38-0.93) p=0.023	0.69 (0.40-1.19) p=0.180
**B. Comparison of HIV incidence among YWSS testing HIV negative at enrolment, by site**				
**DREAMS Site A (n=252)**	16/444.06	3.60 (2.21-5.88)	1.0	
**DREAMS Site B (n=286)**	15/539.70	2.78 (1.68-4.61)	0.75 (0.37-1.53) p=0.431	0.69 (0.33-1.45) p=0.325
**Non-DREAMS Site C (n=121)**	16/227.78	7.02 (4.30-11.47)	1.92 (0.96-3.85) p=0.067	1.55 (0.73-3.27) p=0.252
**Non-DREAMS Site D (n=102)**	11/192.12	5.73 (3.17-10.34)	1.57 (0.73-3.39) p=0.249	1.34 (0.59-3.06) p=0.488
**Non-DREAMS Site E (n=141)**	12/277.64	4.32 (2.45-7.61)	1.21 (0.57-2.56) p=0.615	1.05 (0.46-2.39) p=0.913
**Non-DREAMS Site F (n=115)**	9/210.07	4.28 (2.23-8.23)	1.16 (0.51-2.63) p=0.722	1.01 (0.43-2.35) p=0.984

Supplemental Table 11. Uptake of services available through the DREAMS Partnership, by group (RDS weighted)

	DREAMS cities (N=538) n/N (%)	Non-DREAMS towns (N=481) n/N (%)	DREAMS vs non-DREAMS OR (95% CI)	P-value
**Direct HIV Prevention and Clinical services**				
**Recently HIV tested (within 6mths prior to the survey)**				
No	181/537 (36.6)	152/478 (33.8)		
Yes	356/537 (63.4)	326/478 (66.2)	1.39 (0.81-2.37)	0.234‡
**Ever been offered PrEP**				
No	285/538 (55.5)	476/481 (99.0)		
Yes	253/538 (44.5)	5/481 (1.0)	–	<0.001§
**Current use of contraceptive methods (including condom)**				
No	61/495 (11.8)	101/432 (27.3)		
Yes	434/495 (88.2)	331/432 (72.7)	1.79 (0.81-3.93)	0.148‡
**Attendance to Sisters with a Voice Clinic in past 12 months**				
No	221/538 (43.4)	344/480 (73.0)		
Yes	317/538 (56.6)	136/480 (27.0)	14.54 (7.36-28.75)	<0.001‡
**Saw condom promotion activities in the past 12 months**				
No	175/536 (34.0)	225/479 (48.2)		
Yes	361/536 (66.0)	254/479 (51.8)	1.86 (1.10-3.16)	0.022‡
**Attendance to Sisters with a Voice community mobilisation meeting in past 12 months**				
No	464/537 (86.8)	453/480 (94.3)		
Yes	73/537 (13.2)	27/480 (5.7)	17.24 (2.32-128.20)	0.005‡
**Social and Economic Protection Services**				
**Receipt of cash transfer or educational subsidy in past 12 months**				
No	516/538 (96.1)	480/480 (100.0)		
Yes	22/538 (3.9)	0/480 (0.0)	–	<0.001§
**Participation in continuing education programme in past 12 months**				
No	528/538 (98.2)	480/480 (100.0)		
Yes	10/538 (1.8)	0/480 (0.0)	–	0.018§

**Participation in job preparation training in past 12 months**				
No	529/538 (98.1)	480/480 (100.0)		
Yes	9/538 (1.9)	0/480 (0.0)	–	0.024§
**Participation in apprenticeship in past 12 months¶**				
No	538/538 (100.0)	480/480 (100.0)		
Yes	0/538 (0.0)	0/480 (0.0)	–	–
**Participation in internal savings & loan group in past 12 months**				
No	514/537 (96.1)	479/479 (100.0)		
Yes	23/537 (3.9)	0/479 (0.0)	–	<0.001§
**Gender-based Violence Care and Support Services**				
**Accessed healthcare services after experiencing GBV in past 12 months†**				
No	49/63 (77.4)	40/48 (84.9)		
Yes	14/63 (22.6)	8/48 (15.1)	1.48 (0.20-11.00)	0.701‡
**Provided with shelter in past 12 months (among women experiencing GBV)**				
No	188/189 (99.3)	181/183 (99.3)		
Yes	1/189 (0.7)	2/183 (0.7)	–	0.922§

†Among YWSS who experienced sexual violence
‡Age and site adjusted Wald test p-value
§Fisher's exact p-value – OR and 95% CI could not be estimated using logistic regression due to sparse data
¶Fisher's exact p-value or OR and 95% CI could not be estimated due to sparse data

Chabata ST, *et al. BMJ Global Health* 2022; 6:e003892. doi: 10.1136/bmjgh-2020-003892

Supplemental Table 12. Comparison of DREAMS secondary outcomes between the two DREAMS cities and the four non-DREAMS comparison towns, 2019 (RDS weighted)

		DREAMS cities	Non-DREAMS towns	DREAMS vs non-DREAMS			
		(N=538)	(N=481)	Age-adjusted		Fully-adjusted[‡]	
		n/N (%)	n/N (%)	OR (95% CI)	P-value	OR (95% CI)	P-value
**Improved access to clinical services and HIV prevention services**							
**Knowledge of HIV status**							
	No	119/538 (24.8)	118/481 (25.7)				
	Yes	419/538 (75.2)	363/481 (74.3)	1.07 (0.76-1.51)	0.694	1.12 (0.78-1.60)	0.533
**Ever taken PrEP**							
	No	387/538 (74.4)	478/481 (99.3)				
	Yes	151/538 (25.6)	3/481 (0.7)	47.94 (14.61-157.38)	<0.001	**49.52 (14.62-167.78)**	**<0.001**
**Ability to negotiate condom use with any partner**							
	No	40/538 (7.9)	91/480 (20.7)				
	Yes	498/538 (92.1)	389/480 (79.3)	3.11 (1.95-4.98)	<0.001	**3.68 (2.24-6.04)**	**<0.001**
**Knowledge of the HIV status of at least one of their three most recent partners**							
	No	179/528 (35.8)	210/474 (43.8)				
	Yes	349/528 (64.2)	264/474 (56.2)	1.39 (1.03-1.87)	0.032	1.30 (0.94-1.78)	0.108
**Condom-less sex with regular partner in the past month**							
	No	275/536 (53.0)	199/478 (41.2)				
	Yes	261/536 (47.0)	279/478 (58.8)	0.62 (0.46-0.83)	0.001	**0.65 (0.45-0.93)**	**0.017**
**Condom-less sex with client in the past month**							
	No	475/535 (89.8)	396/478 (83.4)				
	Yes	60/535 (10.2)	82/478 (16.6)	0.57 (0.37-0.85)	0.007	**0.57 (0.35-0.93)**	**0.024**
**Accessed STI treatment services in the past 12 months[§]**							
	No	7/74 (10.0)	18/93 (20.9)				

Chabata ST, *et al. BMJ Global Health* 2022; 6:e003892. doi: 10.1136/bmjgh-2020-003892

Yes	67/74 (90.0)	75/93 (79.1)	2.49 (0.85-7.28)	0.096	–	–
**Improved coverage of Social and Economic Protection service**						
**Food insecurity**						
No	367/538 (70.6)	295/479 (60.7)				
Yes	171/538 (29.4)	184/479 (39.3)	0.65 (0.48-0.88)	0.005	**0.66 (0.48-0.92)**	**0.014**
**Selling sex is the main way to support myself**						
No	196/538 (38.5)	159/479 (36.3)				
Yes	342/538 (61.5)	320/479 (63.7)	0.93 (0.68-1.27)	0.650	0.97 (0.70-1.34)	0.845
**Ever been unable to decline sex in the past month**						
Never/not in the past month	257/534 (50.3)	236/474 (52.6)				
At least once in the past month	277/534 (49.7)	238/474 (47.4)	1.10 (0.82-1.47)	0.513	1.09 (0.80-1.48)	0.586
**Number of sex work clients in the past month**						
≤3	283/537 (56.3)	203/468 (47.0)				
>3	254/537 (43.7)	265/468 (53.0)	0.69 (0.52-0.93)	0.014	**0.63 (0.46-0.87)**	**0.005**
**Gender-based violence prevention, and care and support services**						
**Experience of violence from partners in the past 12 months**						
No	434/538 (83.0)	345/481 (72.6)				
Yes	104/538 (17.0)	136/481 (27.4)	0.55 (0.39-0.77)	0.001	**0.53 (0.36-0.76)**	**0.001**
**Experience of violence from police in the past 12 months**						
No	531/538 (99.1)	473/479 (98.8)				
Yes	7/538 (0.9)	6/479 (1.2)	0.74 (0.20-2.72)	0.652	0.68 (0.18-2.54)	0.565

‡Adjusted for age, highest level of education attained, marital status, self-identification as FSW (measured at baseline), and for each respective secondary outcome measured at enrolment

§OR and 95% CI could not be estimated using logistic regression due to sparse data

REFERENCES

1. AIDS & TB Programme MoHaCC. Zimbabwe national and sub-national HIV estimates report, 2018.

2. Hensen B, Chabata ST, Floyd S, et al. HIV risk among young women who sell sex by whether they identify as sex workers: analysis of respondent-driven sampling surveys, Zimbabwe, 2017. *Journal of the International AIDS Society* 2019; **22**(12): e25410.
